# Targeting inflammation as cancer therapy

**DOI:** 10.1186/s13045-024-01528-7

**Published:** 2024-03-22

**Authors:** Manni Wang, Siyuan Chen, Xuemei He, Yong Yuan, Xiawei Wei

**Affiliations:** 1 Laboratory of Aging Research and Cancer Drug Target, State Key Laboratory of Biotherapy and Cancer Center, National Clinical Research Center for Geriatrics, West China Hospital, Sichuan University, No.17, Block3, Southern Renmin Road, Chengdu, 610041 Sichuan People’s Republic of China; 2 Department of Thoracic Surgery, West China Hospital, Sichuan University, Chengdu, People’s Republic of China

**Keywords:** Inflammation, Cancer, Therapy

## Abstract

Inflammation has accompanied human beings since the emergence of wounds and infections. In the past decades, numerous efforts have been undertaken to explore the potential role of inflammation in cancer, from tumor development, invasion, and metastasis to the resistance of tumors to treatment. Inflammation-targeted agents not only demonstrate the potential to suppress cancer development, but also to improve the efficacy of other therapeutic modalities. In this review, we describe the highly dynamic and complex inflammatory tumor microenvironment, with discussion on key inflammation mediators in cancer including inflammatory cells, inflammatory cytokines, and their downstream intracellular pathways. In addition, we especially address the role of inflammation in cancer development and highlight the action mechanisms of inflammation-targeted therapies in antitumor response. Finally, we summarize the results from both preclinical and clinical studies up to date to illustrate the translation potential of inflammation-targeted therapies.

## Background

Among the key factors contributing to the initiation and progression of tumors, inflammation has been intensively investigated for its supporting role in tumor development. Inflammation has accompanied human beings since the emergence of wounds and infections. The ancient Roman physicians Celsus and Galen described the most prominent evidence of inflammation including “redness, swelling, fever, pain, and dysfunction” [1]. The canonical inflammatory process is characterized by a series of vascular changes, the release of chemicals, and the recruitment of white blood cells to inflammatory sites [2]. In addition to the inflammatory response following wounds and infections, inflammation also exists in other pathologies, such as the chronic inflammation which is known to accompany neurodegenerative diseases, diabetes, atherosclerosis, and most importantly cancer.

In the nineteenth century [3], a German pathologist, Rudolf Virchow brought up a theory that there was certain association between tumor and inflammation as evidenced by leukocyte infiltration. Virchow suggested that tumors might originate from chronic inflammation which persisted though no longer needed. The intratumoral leukocyte infiltration has now become a common hallmark of tumors [4]. In the 1970s, Alexander Haddow proposed that tumor might be caused by “overhealing” of wounds [5]. Given that the development of cancer shares similar features with the tissue regeneration process, Harold F. Dvorak suggested that the inflammatory wound-healing processes might facilitate the generation of tumor stroma [6]. Later in the 1990s, some surgeons reported that operational stress induced by resections could promote angiogenesis which favored tumor growth in nude mice [7].

Tumors are not a simple stack of cells, but rather, consist of heterogeneous cancer cells and stromal cells which collectively provide a complex tumor microenvironment (TME) [8]. Tumors are often characterized with the infiltration of immune cells and the upregulation of inflammatory mediators surrounding tumors. This inflammatory microenvironment may impact tumor development varying stages, from tumor initiation to progression. In this review, we discuss the role of inflammation in cancer development, with special focus on the tumor-promoting activities of inflammation. We especially highlight the underlying mechanisms of the antitumor efficacy of inflammation-targeted therapies in cancer, with clinical evidence up to date in relation to inflammation-targeting strategies.

## Inflammation mediators in cancer

**Fig. 1:**
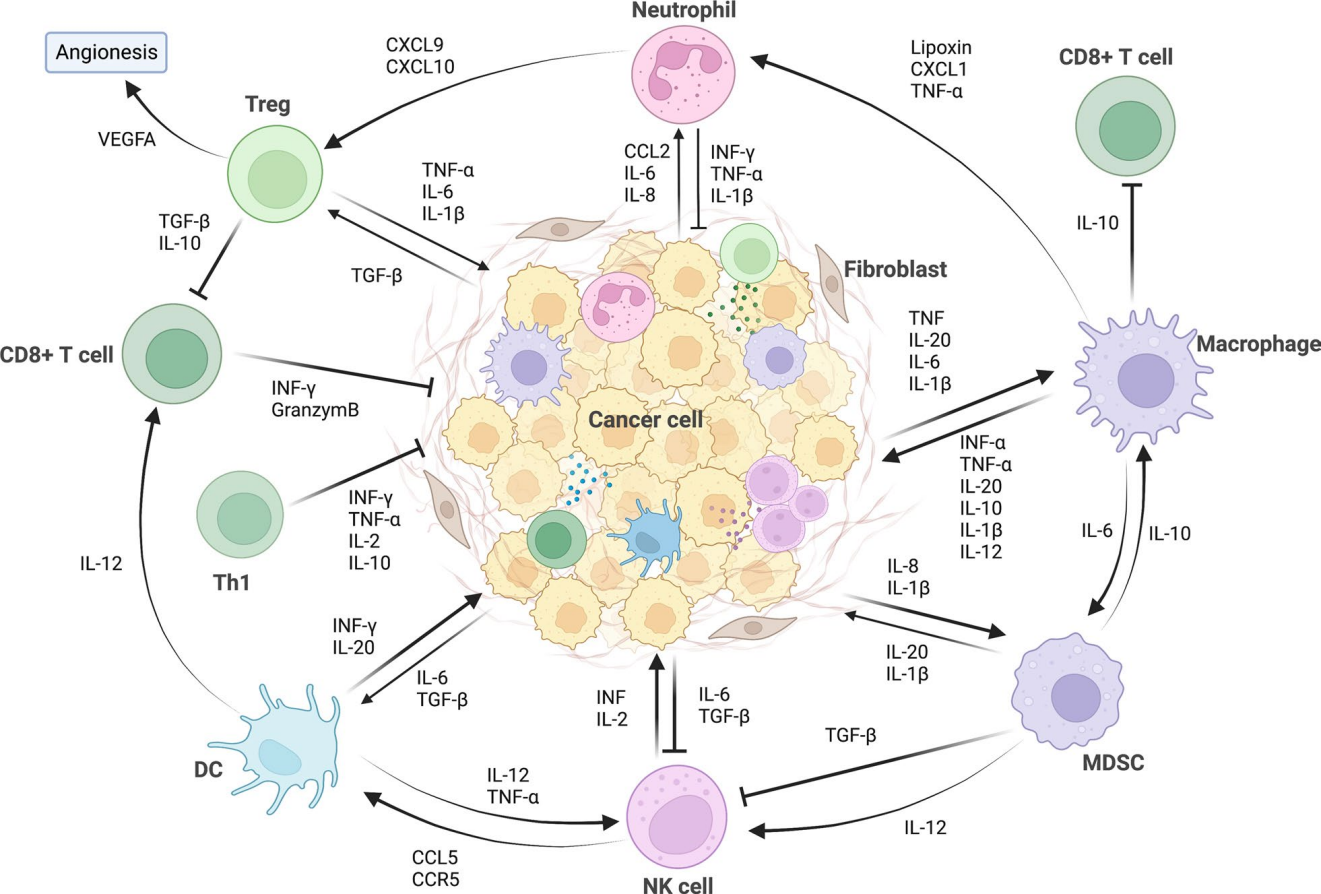
A schematic of the crosstalk between major inflammatory cells and inflammatory molecules in the tumor microenvironment. The major inflammatory cells include T helper (Th1) cell, regulatory T cells (Tregs), cytotoxic CD8 + T cells, macrophages, neutrophils, myeloid-derived suppressor cells (MDSCs), natural killer (NK) cells, and dendritic cells (DCs). Figures created with BioRender. Abbreviations: CXCR, CXC-chemokine receptor; CXCL, chemokine (C-X-C motif) ligand; TGF-β, transforming growth factor-β; TNF, tumor necrosis factor; IL, interleukin; IFN, interferon

The multi-step cancer development process can be initiated by etiologic factors such as carcinogen irritants or oncogenic infection [9]. Under exposure to such etiologic factors, cells with survival advantages transform into tumor-initiating subpopulations with unlimited growth and self-renewal capacity [10]. As demonstrated by epidemiological studies, the ulcerative colitis and Crohn’s disease could increase the risk of colon cancer, which is one of the best known examples of tumor-associated inflammation [11, 12]. Moreover, oncogenic infection by microbial agents such as *Helicobacter pylori *[13] and hepatitis B [14] has also been described as risk factors for gastric and hepatic cancer. During the chronic inflammation induced by microbial agents, immune cells such as macrophages at the inflammatory sites produce reactive oxygen species (ROS), leading to persistent DNA damage and subsequent gene mutations [15]. Furthermore, cytokines secreted by immune cells such as tumor necrosis factor-α (TNF-α) and macrophage migration inhibitory factor (MIF), inhibit the activation of p53- and Rb-E2F pathways and thereby promote tumorigenesis [16, 17]. The various components involved in inflammatory processes form a positive feedback loop that supports cancer progression. The inflammatory cytokines and growth factors then activate transcription factors such as NF-κB, collectively contributing to an inflammatory TME [18, 19]. Figure 1 presents a schematic of the crosstalk between major inflammatory cells and inflammatory molecules in the tumor microenvironment.

### Key inflammatory cells in cancer

The inflammatory TME is highly dynamic and complex, the cell component of which include tumor-associated macrophages (TAMs), tumor-associated neutrophils (TANs), dendritic cells (DCs), myeloid-derived suppressor cells (MDSCs), and T lymphocytes [19]. These tumor-infiltrating cells collectively maintain an inflammatory environment that allows tumor growth and, moreover, immune suppression during tumor progression. The key inflammatory cells involved in cancer with antitumor or protumoral roles are presented in Table 1.

**Table 1:** Key inflammatory cells in cancer with antitumor or protumoral activities

Cell type	Protumor activities	Antitumor activities
**Tumor-associated neutrophils (TANs)**		
	•Promote tumor angiogenesis by inducing continuous release of VEGF from peripheral endothelial cells	•N1 TANs exert an antitumor activity, by direct or indirect cytotoxicity
	•Suppress antitumor immunity via production of proinflammatory	
	•Create immunosuppressive microenvironment via production of immunosuppressive factors	
	•Facilitate the remodeling of local microenvironment that favors tumor cell extravasation through NETs	
**Tumor-associated macrophages (TAMs)**		
	•M2 TAMs induce tumor angiogenesis by upregulating angiogenesis-associated genes such as VEGF	•M1 TAMs facilitate the recruitment and antitumor activities of cytotoxic CD8 + T cells and NK cells
	•M2 TAMs facilitate the degradation of tumor extracellular matrix and the metastasis of tumor cells	
	•M2 TAMs activate the response of endothelial cells to growth factor signaling	
	•M2 TAMs upregulate TGF-β that promotes EMT	
**Dendritic cells (DCs)**		
	•Induce T cell tolerance under pressure of tumor cells	•Provide initial signal for the antitumor response of CD8 + T cells
	•Inhibit the proliferation and functional cytokine production of activated T cells by expressing PD-L1 and PD-L2	•Facilitate antitumor T cell response induced by immunogenic cell death
**Myeloid-derived suppressor cells (MDSCs)**		
	•Suppress antitumor immunity by producing immunosuppressive cytokines	
	•Promote tumor angiogenesis via VEGF and matrix metallopeptidase	
	•Decrease the expansion and activation of tumor-specific T cells by expressing colony-stimulating factor-1 receptor	
**Vascular endothelial cells**		
	•Promote selectin-mediated rolling of tumor cells due to weakened vascular endothelial junctions upon inflammation	•Form a barrier for blood components including tumor cells to infiltrate tissues under physiological conditions

TGF-β, transforming growth factor-β; NETs, neutrophil extracellular traps; NK, natural killer; TAM, tumor-associated macrophage; VEGF, vascular endothelial growth factor

#### Tumor-associated neutrophils (TANs)

Neutrophils constitute the largest proportion of blood leukocytes and are the main population of effector cells upon inflammatory stimuli such as pathogen infection. The N1 and N2 polarization of TANs can be induced by type 1 interferon (IFN) and TGF-β, respectively [20]. Tumor-derived factors induce a shift of infiltrating neutrophils toward an antitumor phenotype [21]. Interestingly, the majority of neutrophils in the TME exhibit an N2 phenotype and facilitate tumor metastasis through various mechanisms [22]. For instance, TANs may promote tumor angiogenesis by inducing continuous release of VEGF from peripheral endothelial cells [23]. In addition, TANs may suppress antitumor immunity by producing various proinflammatory and immunosuppressive factors including IL-1β, IL-17, TNF-α, VEGF, CCL4, matrix metallopeptidase (MMP)-9, C-X-C motif chemokine ligand 8 (CXCL8), and angiopoietin-1 (ANG1) [24]. Known tumor-derived cytokines that drive such differentiation of neutrophils include IFN-γ and GM-CSF which upregulate the expression of specific neutrophil activation markers and thereby promote antitumor activity [25]. Tumor-secreted TGF-β facilitates the recruitment of N2 neutrophils which later creates an immunosuppressive microenvironment by producing CCL2 and CCL17 in a paracrine manner [26, 27]. The increased ratio of TANs to lymphocytes is indicative of poor prognosis in many cancer. The infiltration of TANs and their production of chemokines are able to predict the progression of breast cancer [28].

A unique way for neutrophils to combat infection is the release of neutrophil extracellular traps (NETs), a net-like structure primarily composed of DNA-histone complexes from neutrophils, which are identified as a critical type of innate immune response [29]. Compelling evidence recently suggests that neutrophils can be recruited to the site of pre-metastatic niches such as lung [30], liver [31], and omentum [32] where they facilitate the remodeling of local microenvironment that favors tumor cell extravasation through NETs. The IL-8/CXCL8 autocrine signaling in tumor cells could promote the formation of NETs [33, 34]. Other cancer-induced signals that promote NETs release include CXCR1/CXCR2 agonists, G-CSF, and TGF-β [35–37]. Clinical evidence that linked NETs with cancer was found in Ewing sarcoma, where the presence of intratumoral NETs indicated poor prognosis of patients [38]. The protumorigenic role of NETs may be attributed to their induction of endothelial-to-mesenchymal transition (EMT), an important mechanism for tumor metastasis [39], as observed in models of ovarian [32], lung [40], pancreatic [41], colorectal [42], and breast cancer [43, 44].

However, based on different status of TME, the role of NETs is variable. NETs can also exert an antitumor effect by directly killing tumor cells and inhibiting tumor growth and metastasis. In colorectal cancer (CRC) and head and neck squamous cell carcinoma, in vitro generated NETs could imped tumor growth by inducing apoptosis and inhibiting proliferation [45, 46]. Furthermore, co-culture of melanoma cells with NETs led to necrosis of melanoma cells [47]. NETosis is associated with the release of protein S100A8/A9, the increased ratio of which to CRP was found to correlate with favorable survival of high-grade serous ovarian cancer (HGSOC) patients [48].

#### Tumor-associated macrophages (TAMs)

The wide spectrum of immune functions of TAMs in inflammatory processes such as wound healing has been well documented [49]. Similar to neutrophils, macrophage can also be divided into proinflammatory M1 and anti-inflammatory M2 subtypes [50]. The expression profile of M1 macrophages includes high levels of MHC class II, CD80, and CD86, whereas M2 macrophages highly express CD163 and CD206 [51]. Upon exposure to cytokines such as IL-4, M-CSF/CSF1, IL-10, IL-33, IL-21, and TGF-β, TAMs switch to M2 phenotype, whereas M1 TAMs can be activated by TNF-α or granulocyte–macrophage colony-stimulating factor (GM-CSF), M1 TAMs facilitate the recruitment and antitumor activities of cytotoxic CD8 + T cells and natural killer (NK) cells.

In the inflammatory TME, macrophages account for 30%-50% of cell populations and are believed to provide “soil” for tumor growth. The switch of TAMs between M1 and M2 status largely depends on the molecules present in the TME where tumor cells take advantage of macrophage plasticity to its own benefit ADDIN EN.CITE [52]. At the early stage of the tumor, macrophages polarize to M1 to initiate antitumor responses. When tumors progress to advanced stage, the anti-inflammatory characteristics of TAMs are controlled by tumor cells and polarize to M2 phenotype that promotes tumor progression [53]. M1 macrophages have long been identified as antitumor macrophages, by identifying and directly killing tumor cells. M1macrophage-mediated tumor cell killing is based on its secretion of cytotoxic molecules such as ROS and NO, which is a rather slow process [54]. Another mechanism for M1macrophage-mediated killing of tumor cells is antibody-dependent cell-mediated cytotoxicity (ADCC), which occurs within a few hours and relies on the presence of antitumor antibodies [55]. On the contrary, M2 TAMs are protumoral macrophages that adversely affect the activities of immune effector cells. For tumor healing, the proinflammatory M1 macrophages repolarize into anti-inflammatory M2 TAMs to control inflammation, which unfortunately promote tumor progression [56]. Thus, it is not surprising that a lower M1/M2 ratio of TAMs was significantly related to the progression and poor prognosis of cancer patients [16, 57, 58].

One underlying mechanism for the M2 TAM-induced cancer progression is the direct increase in angiogenesis, mainly by upregulating angiogenesis-associated genes such as VEGF, PDGF, and PGE2 [59]. The indirect proangiogenic effect of the M2 TAMs is mediated by CXCL12, IL-1β, IL-8, and Sema4d which activate the response of endothelial cells to growth factor signaling [60, 61]. M2 TAMs also facilitate the invasion and metastasis of tumors by expressing proteinase, cathepsin, urokinase, and matrix remodeling enzymes which degrade tumor extracellular matrix (ECM) [49]. On the other hand, it was recently reported that miRNAs-containing exosomes released from M2 TAMs could upregulate TGF-β that promotes EMT and causes the imbalance between regulatory T cells (Tregs) and T helper 17 (Th17) cells [62–64]. Moreover, during tumor progression, the presence of M2 TAMs was associated with the malignant potential of tumors and a higher programmed cell death 1 ligand 1 (PD-L1) expression level on tumor and immune cells [65, 66].

#### Dendritic cells (DCs)

DCs are bone marrow-derived cells that detect danger signal in the environment and transmit the signal to adaptive immune cells such as T lymphocytes [67]. Thus, DCs function as a messenger between innate and adaptive immunity. The non-activated DCs are referred to as immature DCs which present self-antigens to T cells, inducing immune tolerance by enhancing the activities of regulatory T cells [68]. DC maturation can be initiated by various signals leading to distinct phenotypes to induce different immune responses, such as fms-related tyrosine kinase receptor 3 (FLT3) [69]. The initial signal for the antitumor response of CD8 + T cells relies on the presentation of tumor-associated antigens (TAAs) on MHC molecules by DCs [70]. In the TME however, the functions of tumor-infiltrating DCs are often suppressed by tumor cells, leading to T cell tolerance rather than antitumor immune response [71]. Presentation of TAAs by DCs in the absence of costimulatory signals may lead to T cell anergy [72]. Tumor-derived factors also modulate the maturation status of DCs, inducing inflammation that favors tumor growth. For instance, tumor-derived IL-6 and M-CSF convert immature DCs into macrophages and prevent the priming of tumor-specific T cells [73]. Furthermore, PD-L1 and PD-L2 expressed on DCs may also inhibit the proliferation and functional cytokine production of activated T cells [74].

In recent decades, immunogenic cell death (ICD) has received considerable research attention. ICD is accompanied by the release and chronic exposure of damage-associated molecular patterns (DAMPs), conferring a potent adjuvanticity to dying cancer cells. ROS production and endoplasmic reticulum (ER) stress are required for the emission of DAMPs which bind to the pattern recognition receptors (PRRs) expressed on immune cells, especially DCs [75]. This recognition and binding process is often associated with the generation of immunological memory [76, 77]. Multiple studies have described the critical role of DCs in the immune response triggered by tumor cells undergoing ICD [78], which demonstrated that the robust antitumor T cell response induced by ICD largely relied on DCs in the TME. It is thus conceivable that manipulating DCs in the TME holds great potential as anticancer strategies. Whereas ICD contributes to the success of many anticancer treatments including chemotherapy, radiotherapy, and target therapies, the immunogenicity varies among cells with different death modalities. A recent study suggested that cancer cells undergoing ferroptosis would impede the maturation of DCs, with poor engulfment and antigen presentation capacity, adding concerns to the applications of ferroptosis-inducing therapeutics [79].

#### Myeloid-derived suppressor cells (MDSCs)

Mouse myeloid-derived suppressor cells (MDSCs) are immature myeloid cells and can be divided into monocytic-myeloid-derived suppressor cells (M-MDSCs) with surface expression of CD11b + Ly6G-Ly6C-high and polymorphonuclear-myeloid-derived suppressor cells (PMN-MDSCs) with CD11b + Ly6G + Ly6C-low [77]. In contrast, the identification of expression profile of human MDSCs is lacking as human leukocytes do not express Gr-1. Given the potent immune-suppressive activities of MDSCs and their similarities with neutrophils and monocytes, it is of paramount importance to identify robust marker combinations and gating parameters for MDSC subsets. A multicenter study identified 10 putative subsets of MDSCs in peripheral blood mononuclear cells (PBMC) obtained from healthy donors to examine the identification marker combinations for circulating MDSCs [80]

The multiple mechanisms for the suppression on antitumor immunity by M-MDSCs have been intensively documented. MDSCs either directly interact with T cells or reshape the TME through the cellular and molecular immunosuppressive network, interfering the normal functions of T cells. M-MDSCs are rapidly recruited to the inflammatory tumor tissues upon exposure to chemokines such as CCL2, CCL5, CXCL8, and CXCL12 and produce multiple immunosuppressive cytokines such as ARG1, nitric oxide (NO), TGF-β, and IL-10 [81, 82]. For example, the upregulation of ARG1 in MDSCs results in L-arginine starvation that leads to T cell dysfunction by decreasing the expression of T cell receptor (TCR) ζ-chain [83]. In addition, MDSC-induced tumor progression is also mediated by tumor angiogenesis. Tumor-derived factors such as VEGF, IL-6, and IL-10 recruit MDSCs which in turn produce more VEGF via STAT3 signaling, thereby establishing a positive feedback loop that potentiates tumor angiogenesis [84, 85]. Apart from the VEGF/VEGFR axis that stimulates MDSCs, the proangiogenic MMPs produced by MDSCs serve as a secondary angiogenetic signals [86]. MMPs are a family of ECM enzymes that facilitate the invasion of tumor cells, and among them MMP9 is perceived as a key regulator for tumor angiogenesis induced by PMN-MDSCs [87].

Given that high M-MDSC fraction is correlated with decreased expansion and activation of tumor-specific T cells [88], MDSCs have now become a novel marker for predicting patients’ response to immune checkpoint blockade (ICB) therapy. For instance, patients with lower fractions of circulating MDSCs are more sensitive to ipilimumab treatment [89], especially melanoma patients [90, 91]. Upon CTLA-4 blockade, tumor-infiltrating MDSCs exhibit increased expression of colony-stimulating factor-1 receptor (CSF-1R), which in turn is correlated with increased MDSC infiltration in tumors. CSF-1/CSF-1R signaling blockade could not only be used to decrease the numbers of MDSCs, but also convert the immune-suppressive MDSCs toward an antitumor phenotype [92, 93]. Likewise, IL-10 secreted by DCs in the TME could increase the number of tumor-infiltrating MDSCs, conferring adaptive resistance to PD-1 antibody treatment [94]. Targeting MDSCs via CSF-1/CSF-1R inhibitors thus becomes a potential strategy to overcome tumor resistance to ICBs. Though a large number of agents targeting the upstream factors or receptors of MDSC accumulation are being tested to potentiate ICB efficacy, it has to be addressed that the majority of MDSC-recruiting chemokines can also act on other immune cells with antitumor activities such as T lymphocytes [95] and NK cells [96]. Thus, such chemokine blockades would possibly yield both positive and negative effect on tumors.

#### Vascular endothelial cells

In addition to immune cells, vascular endothelial cells are also considered a key participant during the inflammatory process in tumors. In direct contact with the cellular and molecular components of blood, vascular endothelial cells form a barrier between blood and the subcutaneous tissue, regulating the permeability of blood vessels and tissue infiltration of blood components. The proinflammatory phenotypes of endothelial cells can be induced by TNF-α and IL-1 released from leukocytes via the TNFR/IL-1 and NF-κB pathway [97]. The activated endothelial cells then express increased luminal endothelial adhesion molecules and produce various chemokines such as CXCL8, CXCL2, complement C5a, leucine, and platelet-activating factor (PAF), mediating the process called vascular inflammation that facilitates leukocyte recruitment into tissues [98]. Due to decreased adhesion molecules upon vascular inflammation, the weakened endothelial junctions make it easier for leukocytes to migrate through vascular walls.

The intricate tumor metastasis process is orchestrated by both cancer and normal cells such as endothelial cells. In the TME, the migration and invasion of cancer cells into tissues are similar to those of leukocytes. However, tumor cells are larger in size and may be mechanically trapped in the blood vessels [99]. To cross endothelial barriers, a large number of molecules such as selectins are required to facilitate leukocyte transmigration [100, 101]. The selectin-mediated rolling of tumor cells represents one of these machinery. For instance the expression of E-selectin on bone marrow endothelial cells and its ligands expression on prostate cancer cells are fundamental for the bone metastasis of prostate cancer [102]. Similarly, E-selectin-mediated rolling of cancer cells on endothelium was observed in breast, pancreatic, and colon cancer [103–105].

### Key inflammatory cytokines in cancer

Cytokines are polypeptides or glycoproteins with molecular weights of less than 30 kDa and could transduce inflammatory or anti-inflammatory signals to cells in the TME. Many of the inflammatory cytokines are associated with the onset and progression of tumors [106], and these cancer-related are often upregulated in the TME [107]. Table 2 presents the key inflammatory cytokines involved in cancer. Understanding the action mechanisms of these cytokines on tumors would facilitate the development of corresponding anticancer therapeutics.

**Table 2:** Key inflammatory cytokines involved in cancer

Inflammatory cytokines	Major sources	Receptors	Key actions in cancer
**TNF-α**	Macrophages, T lymphocytes, NK cells, neutrophils, mast cells, eosinophils and neurons	TNF-αR-1, TNF-αR-2	•Antitumor actions by promoting tumor cell apoptosis, directing TAMs toward the M1 phenotype, and impairing tumor vasculature
			•Promotes the EMT of tumor cells
			•Immunosuppressive actions by promoting Tregs survival and functions
**TGF-β**	Tumor cells, bone matrix	TGF-βRI, TGF-βRII	•Suppresses cancer at early stages of tumorigenesis through apoptosis induction and immune cell modulation
			•Facilitates cancer progression at the later stage by promoting EMT, immune escape, angiogenesis, and suppressing apoptosis
**IFN-I**	DCs, B cells, fibroblasts	IFNAR1, IFNAR2	•Provides proinflammatory signals for tumor progression
			•Facilitates immune evasion of tumor cells
			•Promotes cancer stemness by triggering the epigenetic regulator
			•Antitumor activities by negatively regulating premetastatic niche formation in the TME
**IL-1**	Tumor cells, MDSCs, TAMs, TANs, regulatory B (Breg) cells and Th17	IL-1R	•Promotes tumor progression by recruiting MDSCs to inhibit T cell activation
			•Promotes the production of angiogenic factors such as VEGF by tissue-resident endothelial cells
			•Antitumor activities by inducing Th1-mediated immunity against cancer
**IL-6**	Tumor cells, T cells, B cells, monocytes, fibroblasts, keratinocytes, endothelial cells, mesangial cells, adipocytes	IL-6R	•Promotes tumor progression by inducing tumor cell proliferation, survival, EMT, angiogenesis, and chemoresistance
			•Suppresses tumor cell senescence
**IL-10**	Tumor cells, leukocytes	IL-10R	•Contributes to immunosuppressive microenvironment via exhaustion of intratumoral CD8 + T cells
			•Antitumor activities by promoting the infiltration and cytotoxic activity of CD8 + T cells

TGF-β, transforming growth factor-β; TGF-βR, TGF-β receptor; IL, interleukin; IFN, interferon; TNF, tumor necrosis factor; DC, dendritic cell

#### Tumor necrosis factor alpha (TNF-α)

The regulatory activities of TNF-α in the innate immune system have been reviewed extensively throughout time. TNF-α can be produced by macrophages, T lymphocytes, NK cells, neutrophils, mast cells, eosinophils, and neurons and is involved in a wide range of inflammatory signaling [108]. As a proinflammatory cytokine, the aberrant expression of TNF-α was also identified in multiple malignancies including prostate, ovarian, liver, and breast cancer [109–112]. For instance, the mRNA and protein levels of TNF-α were both upregulated in tumor and stromal cells of breast cancers with worse prognosis [113]. TNF-α is also involved in resistance to anticancer therapy, as evidenced by the decreased sensitivity of gastric cancer to trastuzumab following TNF-α exposure [114]. Strategies targeting TNF-α have been proved effective in pancreatic cancer models [115].

By binding to its receptors TNF-αR-1 and TNF-αR-2, TNF-α promotes tumor proliferation and angiogenesis and induces the EMT of tumor cells [116].

TNF-α may play contrary roles in carcinogenesis depending on its concentrations. The antitumor effect of high concentrations of TNF-α was observed in a murine sarcoma model, whereas low levels of TNF-α led to a protumorigenic phenotype [117].

In melanoma, TNF-α not only induces tumor metastasis ADDIN EN.CITE [118], but also inhibits CD8 T lymphocytes accumulation in the TME ADDIN EN.CITE [119], leading to further evaluation of a TNF-α blockade in pre-clinical models. TNF-α also augments TGF-β signals and promotes TGF-β-induced EMT ADDIN EN.CITE [116]. A recent study suggested that TNF-α upregulates the level of prion protein (PrP) in cancer cells and promotes cancer cell migration ADDIN EN.CITE [120]. TNF-α only exhibits inhibitory effect on Treg functions when in co-culture with effector T cells, but also promotes Treg survival [121]. Several reports suggested that TNF-neutralizing antibodies could increase the Treg frequency in the peripheral blood of patients with rheumatoid arthritis [122, 123]. However, some reports suggested that TNF is able to increase expansion, stability, and possibly function of Tregs via TNFR2 [124]. TNFR2 is highly expressed on Tregs supporting the proliferation and suppressive activities of Tregs [125]. TNFR2 was identified as a expression biomarker for the highly suppressive subset of Tregs [125]. The antagonistic TNFR2 antibodies are thus potential treatment for tumors. TNFR2 antagonists were capable of targeting surface TNFR2 on ovarian cancer cells, inhibiting NF-κB pathway activation and proliferation of tumor cells [126].

#### Transforming growth factor-beta (TGF-β)

Produced by inflammatory cells such as neutrophils and macrophages, TGF-β has long been identified as a pleiotropic cytokine involved in tumor initiation and progression [127]. Three isoforms mammalian TGF-β ligands have been identified so far: TGF-β1, TGF-β2, and TGF-β3, which, by binding to their receptors type I (TGF-βRI) and type II (TGF-βRII), stimulate downstream signaling via phosphorylation of Smads and regulate the transcription of target genes[128]. In addition to tumor cells, the bone matrix is also an important source of TGF-β, linking TGF-β to the bone metastasis of tumors [129].

Interestingly, in the context of tumors, the role of TGF-β may vary according to the stage. In normal condition and early stages of tumorigenesis, TGF-β potently inhibits the growth and development of tumors at the early stage, whereas it induces the proliferation, invasion, metastasis, and angiogenesis of tumors at the later stage [127, 130–132]. The aberrant expression of TGF-β signaling has been found in multiple tumor types including hepatocellular carcinoma, colon, prostate, lung, and breast cancer [133]. Known mechanisms for the TGF-β-mediated tumor support include increased EMT, immune escape, angiogenesis, and suppressed tumor apoptosis [134, 135], whereas the tumor-suppressive role of TGF-β may be mediated by apoptosis induction and immune cell modulation [128]. TGF-β mediates the EMT of tumors potentially by promoting the secretion of MMP2 and MMP9 and suppressing the activity of tissue inhibitors of MMPs (TIMPs) [136]. TGF-β also increases the formation of blood vessels in breast tumors by upregulating VEGF and MCP-1 [137]. It was recently reported that Treg cells work in synergy with tumor cells to create an immunosuppressive TME by secreting TGF-β [138]. Thus, inhibiting TGF-β significantly holds great potential to enhance the efficacy of anticancer treatments.

#### Interferons (IFNs)

IFNs can be classified in type I, type II, and type III based on their structures and receptors and are widely involved in tumor and inflammatory responses. Among them, type I interferons (IFN-Is) consist of 13 isoforms and are widely recognized for their antipathogen and proinflammatory activities. The type I IFN receptor is composed of the IFNAR1 and IFNAR2 subunits. The most important source of type I IFN is plasmacytoid DCs (pDCs) which are also referred to as the natural “IFN-producing cells.” In addition, B cells are also able to produce type I IFN in vivo, and fibroblasts can produce IFNβ upon after viral infections [139, 140]. In recent decades, emerging data suggest that IFN I is implicated in many aspects of antitumor immunity such as antigen presentation, tumor cell apoptosis, and immunosuppression.

During chronic inflammation, the feedback protective processes induced by IFN-Is provide tumor cells with supportive microenvironment for tumor growth and progression [141, 142]. Alongside the proinflammatory signals for tumor progression, IFN-Is may also facilitate the immune evasion of tumor cells by upregulating immune-suppressive pathways ranging from danger sensing to cytokine production [143, 144]. For instance in head and neck squamous cell carcinoma (HNSCC), cancer-specific IFN-I activation attenuates the expansion and functions of CD8 + T effector cells and is associated with poor clinical outcomes [145].

In addition, IFN-I was reported to promote cancer stemness by triggering the epigenetic regulator KDM1B [146]. IFN-stimulated genes (ISGs) are overexpressed in epithelial cells which spontaneously trigger EMT of tumor cells, thereby regulating EMT and subsequent tumor metastasis at multiple levels [147]. However, studies have also delineated the antitumor activities of IFN-Is which negatively regulate premetastatic niche formation in the TME [148]. Further, the potent antiangiogenic activity of IFN-Is especially IFN-α has been reported [149]. IFN-α was approved for the treatment of hairy cell leukemia in 1986 [150]. A growing body of literature then investigated the efficacy of IFNs in both hematological malignancies and solid tumors. Thus, the role of IFN-Is in cancer may be highly dependent on cell type, timing, and various other factors.

#### Interleukin-1

Interleukin (IL)-1 is upregulated in multiple tumor types including breast, colon, head and neck, lung, pancreas cancer, and melanomas, the high expression of which is indicative of bad prognosis [151]. The endogenous IL-1 produced by cancer cells acts as a growth factor that promotes the synthesis of other cytokines such as IL-6 and TGF-β in a paracrine and autocrine manner [152, 153]. It was recently reported that the baseline IL-1 expression and the newly produced IL-1 in response to CD40 agonists are both correlated with the resistance of in melanomas to immunotherapy [154]. Positive correlations were identified between IL-1β expression and the infiltration of immunosuppressive MDSCs, as well as the expression of their chemoattractants in patients with K-ras-mutant lung adenocarcinoma (KM-LUAD), suggesting the therapeutic potential of IL-1β blockades. However, some studies presented different results that supported the antitumor role of IL-1. For example, IL-1 has been found to induce Th1-mediated immunity against cancer [155]. Such dual activities of IL-1 in cancer require more detailed assessment when developing therapeutic intervention strategies targeting IL-1 [156].

In the TME, immunosuppressive cells including MDSCs, TAMs, TANs, regulatory B (Breg) cells, and Th17 are a major source of IL-1, which also are in turn regulated by IL-1 [157]. IL-1 plays a pivotal role in the differentiation of Th17 cells from naïve T cells and facilitates the maintenance of Th17 cell phenotypes [158]. Tumor-released IL-1α promoted tumor development by recruiting MDSCs to inhibit T cell activation [159]. The elevated level of IL-1β in the serum of advanced melanoma patients was associated with higher frequency of MDSCs and Tregs [160]. In addition, MDSC-secreted IL-1β promotes the production of angiogenic factors such as VEGF by tissue-resident endothelial cells [161, 162]. The immunosuppressive TME provides rationale for the combinatorial use of checkpoint blockades and IL-1 inhibitors, which displayed a synergistic antitumor effect in a breast cancer mouse model [163]. Similar results were reported in pancreatic ductal adenocarcinoma (PDAC) model where IL-1β blockade sensitized tumors to the PD-1 blockade [164].

#### Interleukin-6

Interleukin (IL)-6 is a family of protumorigenic cytokines consisting of IL-11, IL-27, IL-31, leukemia inhibitory factor (LIF), oncostatin M (OSM), ciliary neurotrophic factor (CNTF), cardiotrophin-1 (CT-1), and cardiotrophin-like cytokine (CLC), the role of which has been well characterized in the regulation of tumor growth and metastasis. IL-6 can be produced by multiple cell types including T cells, B cells, monocytes, fibroblasts, keratinocytes, endothelial cells, mesangial cells, adipocytes, and tumor cells. By interacting with IL-6 receptor (IL-6R), IL-6 activates STAT3 by upregulating the expression of cyclin D1, D2, and B1, and c-Myc and downregulating the expression of the cyclin-dependent kinase (CDK) inhibitor p21, which collectively accelerates the entry of tumor cells into cell cycles [165]. Moreover, tumor cells partially rely on the IL-6/STAT3 axis to escape cell death induced by cytotoxic drugs. IL-6-activated STAT3 in turn promotes tumor cell survival by inducing the expression of Bcl-2, survivin, and X-linked inhibitor of apoptosis protein (XIAP), the overexpression of which is related to increased chemoresistance [166, 167]. IL-6 may also contribute to cell proliferation, survival, and chemoresistance of tumor cells by activating the Ras-ERK and PI3K-Akt pathways [168]. Other mechanisms for the protumorigenic effect of IL-6 include the suppression of tumor senescence [169, 170], the interaction with growth factor signaling [171], the induction of EMT [172, 173], and angiogenesis [174]. Notably, IL-6 has been found to be overexpressed in common metastatic organs such as lung, liver, brain, and bone marrow, which is conductive to the seeding of circulating tumor cells to establish metastatic lesions [175–177].

#### Interleukin-10

IL-10 was initially conceived as a secreted cytokine synthesis inhibitory factor, known to inhibit cytokine production of Th1 cells [178] and activate macrophages and DCs [179, 180]. As a key mediator of the anti-inflammatory response, IL-10 family cytokines are mostly produced by leukocytes, as well as human tumor cells. This cytokine family consists of IL-10 and IL-20 subfamily cytokines including IL-19, IL-20, IL-22, IL-24, and IL-26 [181]. IL-10 suppresses uncontrolled inflammatory responses, thereby maintaining homeostasis [182]. In tumors such as gastric cancer, TAM-produced IL-10 contributes to an immunosuppressive microenvironment that favors tumor growth [183]. A more recent study showed that the expression of IL-10 in tumor-infiltrating regulatory T cells may result in the exhaustion of intratumoral CD8 + T cells [184]. Some studies on the other hand suggested that IL-10 can be used as an immunotherapy in tumor models [185]. IL-10 could induce the expression of CD3 and CD8 molecules on thymocytes and thereby promotes the cytotoxic activity of CD8 + T cells [186]. Another mechanism for the antitumor action of IL-10 is the increased CD8 + T cell infiltration and IFN-γ level in tumor tissues induced by IL-10 [181]. The discrepancies may be attributed to the tumor types or different stages of T cells that respond to IL-10. It is thus critical to assess the context before determining the either protective or detrimental role of IL-10 in cancer therapy.

#### ROS

Reactive oxygen species (ROS) are a large family of reactive molecules, including hydrogen peroxide (H2O2), hydrogen radicals (·OH), hydroxyl ions (OH −), superoxide anions (·O2 −), singlet oxygen (1O2), nitric oxide (NO −), peroxynitrites (ONOO −), and hypochlorite (OCl −) [187]. ROS are capable of rapidly switching one specie to another through cascade reactions because they are equipped with. Due to their unpaired valence electrons and unstable bonds, ROS rapidly switch from one to another and are therefore short-lived. As an essential signal molecule, ROS is implicated in various physiological possess, whereas excessive generation of ROS is associated with oxidative stress overload, leading to cell dysfunction and inflammation [188, 189]. Mitochondria are the major source of ROS and are actively involved in oxidative phosphorylation chain [190]. During aberrant oxidative phosphorylation, electrons escape and react with O2 to produce superoxide anions, which are then converted to H2O2 in the mitochondrial matrix. It has to be addressed that not all mitochondria-produced ROS derive from oxidative phosphorylation, with approximately 30% of H2O2 generated from oxidation of cytochrome C [191], and recently reported to be generated from nicotinamide adenine dinucleotide phosphate (NADPH) oxidase [192]. Glutathione peroxidase (GPx) represents another endogenous antioxidant mechanism which degrades hydroperoxides [193]. In addition, the external stimuli such as chemotherapy, radiotherapy, and ultraviolet may also trigger ROS production [194].

Cancer cells carry higher amount of ROS than their normal counterparts, due to aberrant oncogene activation and mitochondrial activity. The role of ROS in cancer development is intricate, making it a double-edged sword [195]. On one hand, the sustained ROS stress may damage cell structures, impede their biological functions, and cause mutagenesis, which collectively increase the risks for oncogenesis [196, 197]. On the contrary, ROS may accumulate upon exogenous stimuli such as chemotherapy and radiotherapy, leading to tumor cell death and thereby sensitizing tumor cells to treatments. Elucidating the complex roles of ROS in cancer will aid the design of ROS-targeting therapies for cancer. Recent studies suggest that hypoxic environment in tumors could activate ROS generation [198]. In response to hypoxia, the hypoxia-inducible factor-1 (HIF-1) is a well-characterized transcriptional activator that modulates oxygen homeostasis [199]. By interacting with hypoxia response elements of target genes, ROS promotes the activation of HIF-1α, leading to subsequent transactivation of genes that augment hypoxic adaptation [200, 201]. It was recently reported that hypoxia-induced ROS augment the hypoxic adaptation of glioblastoma by mediating the HIF-1α-SERPINE1 signaling pathway, making ROS a promising therapeutic target for glioblastoma [202].

### Key inflammatory pathways in cancer

Despite the cellular components of cancer-related inflammation, the vast majority of regulatory molecules have been identified to facilitate the protumorigenic effect of inflammation. Such molecules range from inflammatory cytokines to their downstream target molecules and transcription factors, represented by the eicosanoid signaling, and the Janus kinase (JAK)-signal transducer and activator of transcription (STAT) signaling.

#### Eicosanoid signaling

Eicosanoids are highly bioactive oxidized derivatives of 20-carbon polyunsaturated fatty acids (PUFAs) that can be produced through the cyclooxygenase (COX), lipoxygenase (LOX), and cytochrome P450 (cytP450) pathways. Whereas the COX pathway produces prostaglandins (PGs) and thromboxanes (TXs), the LOX pathway is known to generate leukotrienes (LTs) and lipoxins (LXs) [203]. The rapid catabolism of eicosanoids constrains their activities to the local sites of their production [110]. The eicosanoid signaling cascades play a pivotal role in both physiological processes and pathological processes such as tumorigenesis.

##### Cyclooxygenase (COX) signaling

The COX pathway is a well-studied mechanism through which eicosanoids are formed and link inflammation with cancer. COX-1 and COX-2 are two key isoforms of COX enzymes. Under physiologic conditions, the constitutive expression of COX-1 is important for maintaining tissue homeostasis. On the other hand, the expression of COX-2 is upregulated by proinflammatory stimuli. Another isomer COX-3 has recently been identified, the function of which remains to be further elucidated [204, 205]. Among them, COX-2 has been intensively studied for its regulation of cancer-associated inflammation and cancer progression. The upregulation of COX-2 was first identified in human colorectal adenomas and adenocarcinomas [206] and was found to correlate with inflammatory bowel disease and colorectal cancer [207]. The association between COX-2 overexpression and unfavorable prognosis has later extended to various cancer types including melanoma [208], breast [209], prostate [210, 211], laryngeal [212], esophageal [213], gastric [214], pancreatic [215], and ovarian cancer [216].

During the early stage of the inflammatory response, COX-2-derived PGs are assumed to display proinflammatory functions [217]. The prostaglandin D2, prostaglandin E2, prostaglandin F2α, prostaglandin I2, and thromboxane A2 are five key PGs derived via the COX pathway. Among them, PGE2 is the most common prostaglandin in cancer, the upregulation of which is associated with poor prognosis and more advanced tumor stage [218–220]. Accordingly, genetic deletion of microsomal PGE2 synthase 1 (mPGES-1) gene leads to decreased intestinal tumor growth by 66–95% [221]. Furthermore, PGE2 may also promote tumorigenesis by inducing immune suppression [222, 223]. PGE2 potently regulates IFN-γ synthesis of NK cells, which is an important proinflammatory event [224]. The MDSCs were found to express receptors for PGE2, the antagonists of which could block the differentiation of MDSCs [225]. PGE2 may enhance the immunosuppressive phenotype of mononuclear (M)-MDSCs and potentiate its inhibitory activities on T cell proliferation [226]. In response to IFN-γ, tumor-derived PGE2 also induces nuclear p50 NF-κB that epigenetically reprograms monocyte toward an immunosuppressive phenotype, providing another rationale for the tumorigenic effect of PGE2 [227].

In contrast to prostaglandin E2 the role has been established in cancer, prostaglandin D2, another COX-2 metabolite and may play dual roles in chronic inflammation and cancer. The interaction between PGD2 and its receptor PTGDR2 inhibits the self-renewal of gastric cancer cells and attenuates the growth and metastasis of gastric tumors [228]. In addition, PGD2 also inhibits colitis and colitis-associated colon cancer in mouse models [229]. It was recently reported that PGD2 could reduce the proliferation of lung cancer cells, but at the same time enhance their invasion and migration [230], leading to the hypothesis that the exact role of PGD2 in cancer may vary according to the tumor stage.

**Fig. 2:**
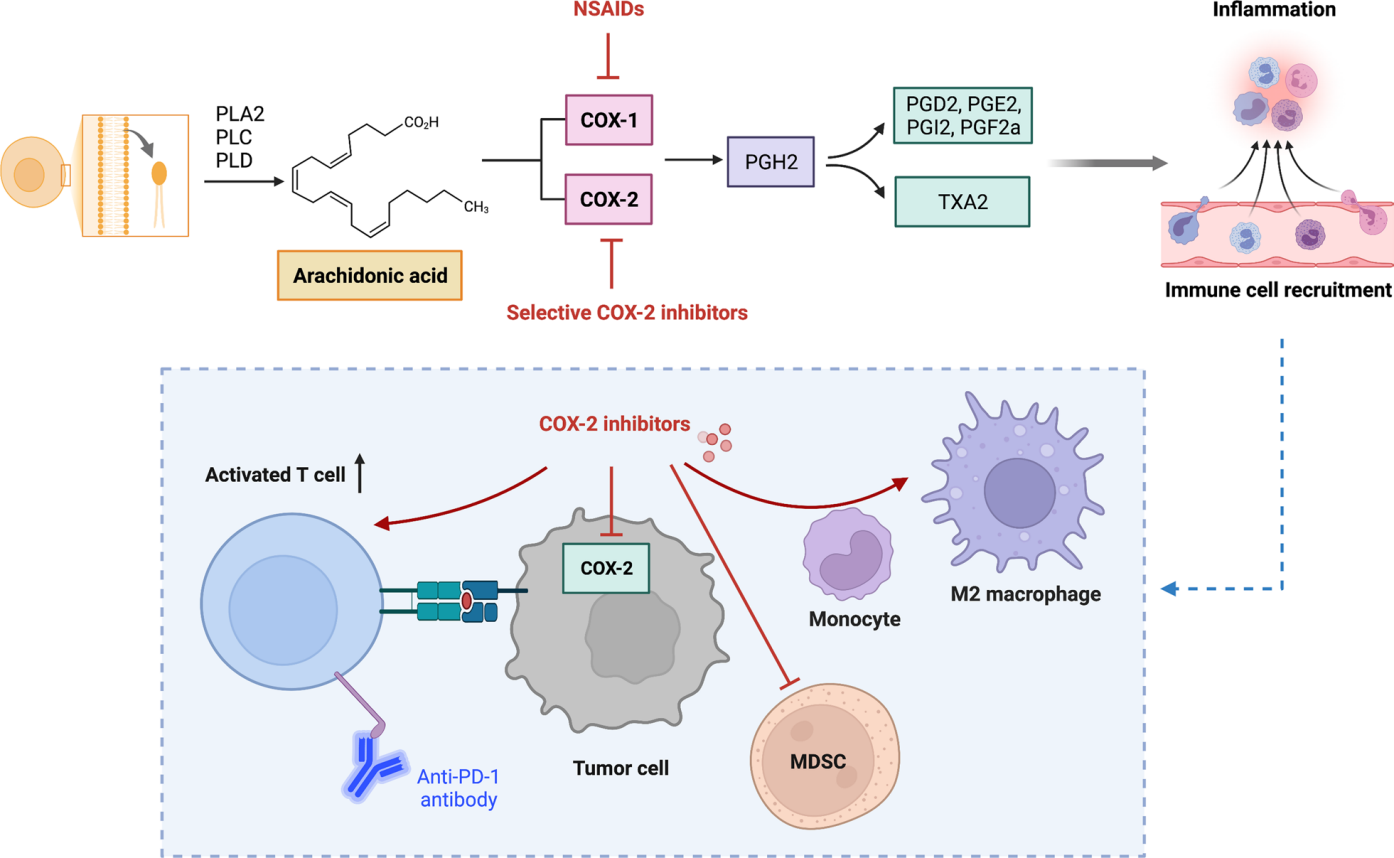
Overview of the cyclooxygenase pathway and the action mechanisms of cyclooxygenase-targeting strategies in cancer. The COX-2/PGE2 pathway facilitates the maintenance of immunosuppressive TME by activating a wide range of immunosuppressive immune cells. Inhibitors of COX-2 signaling such as NSAIDs are potentially a good combination partner for immunotherapies. Figures created with BioRender. Abbreviations: PGH2, prostaglandin H2; PGG2, prostaglandin G2; PLA2, PLC, PLD, phospholipases A2, C, and D; PGE2, prostaglandin (PG) E2; PGI2, prostacyclin; PGD2, prostaglandin D2; PGF2α, prostaglandin F2α; TXA2, thromboxane A2; MDSC, myeloid-derived suppressor cells

The contributing role of COX-2/PGE2 in immunosuppression has long been studied even before the advent of immunotherapy. The association between COX-2 expression and T cell exclusion was found in pancreatic cancer models [231]. The intrinsic TGF-β signaling of pancreatic tumor cells induced the overexpression of PTGS2, leading to decreased level of activated CD8 + T cells in the TME [231]. In addition, COX-2/PGE2 signaling is associated with the accumulation of MDSCs. Thus, blocking COX-2/PGE2 signaling could reshape TME by reversing the immunosuppressive activities of MDSCs [232]. Moreover, PGE2 also impacts the polarization status of macrophage by inducing monocyte differentiation into the M2-like macrophage [233]. Given that the COX-2/PGE2 pathway facilitates the maintenance of immunosuppressive TME by activating a wide range of immunosuppressive immune cells, inhibiting COX-2 signaling is potentially a good combination partner for immunotherapies, such as checkpoint inhibitors (Fig. 2).

##### Lipoxygenase (LOX) signaling

The LOX pathway mainly comprises 5-LOX, 12-LOX, and 15-LOX [110]. Whereas 5-LOX and 12-LOX have been identified with angiogenetic and protumorigenic activities, 15-LOX exerts both protumorigenic and antitumorigenic effects [234]. As a key enzyme in metabolizing arachidonic acid to leukotrienes, 5-LOX is highly expressed in epithelial cancers as well as lymphomas [235, 236]. Inhibiting approaches targeting 5-LOX were used to inhibit tumorigenesis [226, 237]. Given that both 5-LOX and COX-2 are upregulated in inflammation-related tumors, the concomitant inhibition of 5-LOX and COX-2 was designed to render more potent tumor suppression than inhibition of a single eicosanoid pathway [116, 238, 239].

The 12-LOX is a key enzyme that mediates the generation of 12-HETE which in recent years has been identified to facilitate tumor growth by activating the integrin-linked kinase/NF-κB pathway [240, 241]. 15-LOX-1, on the other hand, can be expressed in Hodgkin lymphoma cells, and its metabolites were found to enhance tumor-associated inflammation [242]. As discussed earlier, 15-LOX may have antitumorigenic role in cancer. A recent study suggested decreased levels of 15-LOX in doxorubicin (DOX)-resistant cells compared with their DOX-sensitive counterparts. The overexpression of 15-LOX could induce DOX accumulation in DOX-resistant breast cancer cells and promote their apoptosis [243]. Similar data were obtained from colorectal cancer (CRC) model where deficient 15-LOX-1 was correlated with the radioresistance of CRC cells, potentially by downregulating the histone H2A variant macroH2A2 [244].

The LOX pathways are responsible for metabolizing arachidonic acid to leukotrienes such as leukotriene A_4_ (LTA_4_) and leukotriene B_4_ (LTB_4_). Inflammatory cells including leukocytes, macrophages, and mast cells are the major source of leukotrienes [245]. LTB_4_ was found to promote inflammation-induced melanoma, and the inhibition of LTB_4_ receptors may suppress the progression of inflammation-associated tumors [246]. The leukotriene D_4_ (LTD_4_), derived from the 5-LOX-catalyzed oxygenation of arachidonic acid, is upregulated in the circulation of patients with hepatocellular carcinoma and chronic hepatitis B [247, 248]. Recent studies investigated the efficacy of leukotriene receptor antagonists as a novel combination partner for conventional multi-kinase inhibitors in the treatment of hepatic cancer [249].

On the contrary, another LOX-derived eicosanoids, lipoxins (LXs), are characterized as antitumorigenic [250]. Lipoxins stimulate monocytes without causing the inflammatory release of ROS [251]. Lipoxins may also promote the phagocytosis of apoptotic neutrophils by macrophages, thereby reducing inflammation[252]. Accumulating evidence suggests the anti-inflammatory effect of lipoxin A_4_ (LXA_4_) in inflammation-associated cancers such as colorectal cancer [253]. In prostate cancer, LXA_4_ promotes the M2 polarization of macrophages by inhibiting METTL3 [254]. Other mechanisms for the LXA4-induced polarization of M2 macrophages may be mediated via the FPR2/IRF4 pathway [255]. However, a recent study reported that lipid mediators such as lipoxins could induce the angiogenesis, proliferation, and treatment resistance of glioblastoma cells [256]. More studies are warranted to elucidate the potential of endogenous lipoxin administration in combating cancer.

#### JAK-STAT signaling

The JAK/STAT signaling is a highly conserved pathway with the ligand–receptor interaction machinery. The JAK family consists JAK1, JAK2, JAK3, and TYK2, and the STAT family members include STAT1, STAT2, STAT3, STAT4, STAT5a, STAT5b, and STAT6 [257]. In general, the receptors–ligand interaction induces the phosphorylation of JAKs which then form a docking site for STATs leading to STAT phosphorylation. As the core member of the STAT protein family, STAT3 plays a with versatile roles in the inflammatory response and tumor progression. Multiple growth factors and cytokines are implicated in the canonical STAT3 pathways, regulating the transcription of STAT3 target genes and downstream cellular processes such as cell differentiation, angiogenesis, and tumorigenesis [258]. The dysregulated STAT3 signaling has been implicated in a series of inflammatory diseases such as rheumatoid arthritis, multiple sclerosis, and inflammatory bowel disease [259]. Moreover, the persistent activation of the STAT3 signaling may result in the tumorigenesis of both solid and hematological malignancies [260].

Chronic inflammation is a key event of tumorigenesis [261]. Genome-wide association studies have identified a certain correlation between STAT3 and the susceptibility to inflammatory bowel disease (IBD) [262]. Cytokines that induce the activation of STAT3 are upregulated in IBD such as IL-1β, IL-6, IL-12, IL-15, IL-10, IFN, and TNF-α [263]. It has been well established that IL-6 and STAT3 are required for survival and proliferation of tumor-initiating intestinal epithelial cells [264]. As a critical regulator of the inflammatory process, the IL-6/STAT3 signaling is implicated in inflammation-associated tumors such as CRC and colitis-associated CRC (CAC) [265]. Furthermore, in CRC stroma, cancer-associated fibroblasts (CAFs) produce IL-6 which upregulates the expression of metastasis-associated markers such as Leucine Rich Alpha-2-Glycoprotein 1(LRG1) via the JAK2/STAT3 signaling [266].

The status of the gut microbiome which metabolizes bile acid in the intestine is another important determinant of intestinal inflammation, with certain microbes either promoting or suppressing tumorigenesis of CRC [267]. The loss of integrity of intestinal epithelial barriers and the recognition of PAMPs by PRRs leads to increased secretion of inflammatory factors that activate STAT3, thereby evoking inflammatory response in CRC. Similar results were observed in prostate cancer where gut dysbiosis increased gut permeability and intratumoral LPS which promotes tumor progression via NF-κB/IL6/STAT3 axis [268].

#### Metal metabolism

Iron is indispensable for multiple cellular events such as cell survival and biological processes such as oxygen transport and deoxyribonucleic acid (DNA) synthesis [269]. Dysregulated iron metabolism is a crucial hallmark of tumor cells where malignant cells need substantial amount of iron to survive and proliferate. In the Fenton reaction, the redox-active iron (Fe2 +) reacts with H2O2 which directly generates ferric iron (Fe3 +) and a large amount of hydroxyl radicals [270]. As aforementioned, the balance between ROS generation and detoxification is important to prevent the oxidative stress and ROS-mediated cell death [271]. Iron-dependent enzymes such as cytochrome P450 enzymes, nitric oxide synthases, NADPH oxidases, and lipoxygenases are involved in the generation of ROS [272]. Excessive iron is also associated with ferroptosis, a type of regulated cell death. GPX4 is the key regulating glutathione peroxidase of ferroptosis, which converts lipid hydroperoxides to lipid alcohols, and prevents the iron (Fe2 +)-dependent formation of ROS [273]. Thus, inhibiting GPX4 could enhance the antitumor response of therapies by inducing ferroptosis. Nevertheless, even with high oxidative stress, ferroptosis is not a frequent event in tumor cells. Several agents have been identified with ferroptosis-inducing capacity, including erastin, a voltage-dependent anion channels (VDAC)-2/3 inhibitor, and sorafenib, a multikinase inhibitor [274].

Zinc is the second most abundant fundamental nutritional element in human body, which was first documented in the 1960s regarding its role in human health [275]. Zinc is implicated in the production and signaling of numerous inflammatory cytokines, and upon acute response to stress stimuli, plasma concentrations of zinc rapidly drop. Zinc metabolism in humans is tightly associated with the activities of zinc transporters such as ZIP8. During inflammation, activated NF-κB increases the expression of ZIP8 which localizes to cell membrane and regulates zinc uptake. Following the entry of zinc into cytosol, zinc suppresses IKKβ activities and thereby attenuates the inflammatory response, all of which form a negative feedback loop [276]. These results highlight the regulating role of metal metabolism in inflammation and cancer and unveil the therapeutic potential of metabolic reprogramming in disease treatment.

## Inflammation-targeted therapies in cancer

**Fig. 3:**
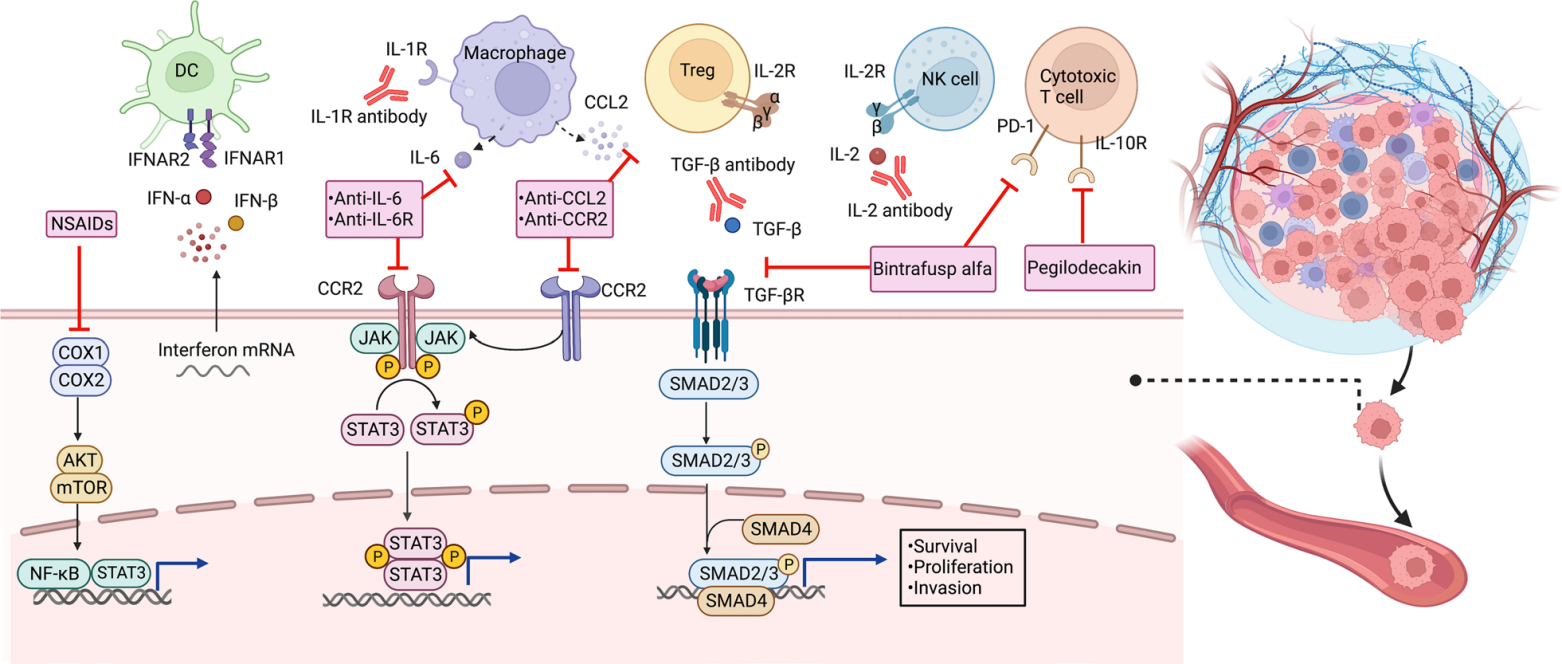
Molecular mechanisms that mediate the effects of inflammation-targeting strategies in cancer. These inflammation-targeting strategies inhibit the COX, JAK/STAT, and TGF-β signaling which support cancer cell survival, proliferation, and invasion. Figures created with BioRender. NSAIDs, non-steroidal anti-inflammatory drugs; COX, cyclooxygenase; mTOR, mammalian target of rapamycin; NF-κB, nuclear factor kappa B; CXCR, CXC-chemokine receptor; CXCL, chemokine (C-X-C motif) ligand; TGF-β, transforming growth factor-β; TGF-βR, TGF-β receptor; IL, interleukin; IFN, interferon; STAT3, signal transducer and activator of transcription 3; SMAD, mothers against decapentaplegic

As aforementioned, the inflammatory cells and mediators including cytokines, chemokines, and eicosanoids form an intricate network in the TME and regulate tumor-associated inflammatory responses. Emerging preclinical results have motivated the design of anti-inflammatory agents for the treatment of cancer, either as monotherapy or in combination with other therapeutic modalities (Table 3). We herein discuss the current application of inflammatory-targeted treatments and the potential for translating current knowledge on cancer-related inflammation into clinical practice. The molecular mechanisms that mediate the effects of inflammation-targeting strategies in cancer are presented in Fig. 3.

**Table 3:** Key anti-inflammatory agents tested in clinical trials in cancer

Agent/target	Tumor type	Combination regime	Key clinical trial	Reported action
**Celecoxib**				
COX-2	Breast cancer	Neoadjuvant celecoxib + chemotherapy/cholecalciferol/exemestane	NCT02429427, NCT01041781	Celecoxib induced favorable changes in serum biomarkers and cytology in women with increased risk for breast cancer, but demonstrated no significant benefits for patients with ERBB2-negative breast cancer
	Lung cancer	Celecoxib + chemotherapy/RT/anti-EGFR TKIs	NCT00300729, NCT01503385	Celecoxib at a maximal tolerated dose of 800 mg/d can be safely administered concurrently with thoracic radiotherapy of NSCLC
	CRC	Celecoxib + cetuximab/chemotherapy (FOLFIRI regimen)/RT/	NCT03645187, NCT00005094, NCT00141193, NCT03926338, NCT01150045	Celecoxib combined with chemotherapy (FOLFIRI regimen consisting of 5-flourouracil, leucovorin, irinotecan) or PD-1 blockade toripalimab represents an effective and safe synergetic protocol for patients with metastatic CRC
**Antiviral therapies**				
**Entecavir**				
HBV	HCC		NCT00388674	Entecavir led to a reduced risk of HBV-related events including HCC
**Tenofovir**				
HBV	HCC		NCT019553458	Tenofovir led to a comparable long-term risk of HCC and ICC in CHB patients with entecavir
**ISA 101 HPV-16 vaccine**				
HPV	Cervical cancer	ISA 101 + anti-PD-1 antibody nivolumab	NCT02426892	Concurrent treatment of ISA 101 and anti-PD-1 antibody nivolumab increased both overall response rates and survival of HPV-16-related cancer
**Cytokine-directed therapies**				
**IFN-α**	RCC	IFN-α + oblimersen/(iso)tretinoin/isotretinoin/IL-2/chemotherapy (fluorouracil, capecitabine)/sorafenib/VEGF inhibitor (bevacizumab, SU5416)/mTOR inhibitor (CCI-779)/naptumomab estafenatox/pazopanib/celecoxib/thalidomide/chemotherapy (5-Fluorouracil) /pembrolizumab	UMIN000002466, CALGB 90206	The prolonged IFN-α treatment induced long-lasting complete responses and long-term outcome with acceptable toxicity in patients with metastatic RCC. IFN-α is also a promising combination therapy for target therapies and immune checkpoint inhibitors such as anti-PD-1 therapies
	Melanoma	IFN-α + combination chemotherapy (dacarbazine, temozolomide, azacitidine, cisplatin)/IL-12/thalidomide/bevacizumab/imatinib/BRAF inhibitor (vemurafenib)/CTLA-4 inhibitor ipilimumab/proteasome inhibitor (PS-341)/sodium stibogluconateRT	NCT00204529, NCT01959633, EORTC 18991, S0008	Adjuvant treatment with IFN-α-2a or PEG-IFN-α-2b could induce sustained improvement of RFS in stage III melanoma patients and has been approved by the FDA as adjuvant therapy for melanoma
	Leukemia	IFNα-2a + combination chemotherapy (melphalan, adriamycin, bleomycin, velban, and dacarbazine)/nilotinib/imatinib/rituximab/dasatinib	NCT02328755, NCT02185261	IFN-α treatment is an effective strategy for minimal residual disease (MRD)-positive leukemia patients receiving allogeneic hematopoietic stem cell transplantation (allo-HSCT)
	Lymphoma	IFN-α + combination chemotherapy (melphalan, adriamycin, bleomycin, velban, and dacarbazine)/bexarotene/rituximab	NCT01609010	Immunotherapy with IFN-α and rIL-2 is well tolerated and may intensify remission in NHL patients
	HCC	IFN-α + chemotherapy (capecitabine)/celecoxib + rintatolimod/thalidomide		IFN-α therapy may reduce HCC recurrence after medical ablation therapy for primary tumors. IFN-α plus cis-platinum is effective in patients with inoperable HCC
**Galunisertib (LY2157299)**				
TGF-β	Pancreatic cancer	Galunisertib + durvalumab/gemcitabine	NCT02734160	The galunisertib-gemcitabine combination improved OS in patients with unresectable pancreatic cancer with minimal added toxicity
	HCC	Galunisertib + sorafenib/stereotactic body radiotherapy (SBRT)	NCT01246986	The combination of galunisertib and sorafenib demonstrated a manageable safety profile and improved prognosis of HCC
**Fresolimumab (GC1008)**				
TGF-β	Melanoma, RCC		NCT00356460	Fresolimumab displayed preliminary antitumor efficacy and acceptable safety profile at multiple doses in patients with advanced melanoma and RCC
**PF-03446962**				
TGF-β	HCC, CRC	Regorafenib + PF-03446962	NCT00557856	PF-03446962 had manageable safety and pharmacokinetic profiles in HCC, but the combination of regorafenib and PF-03446962 caused unacceptable toxicity with limited clinical activity in patients with refractory metastatic CRC
**Bintrafusp alfa (M7824)**				
TGF-β and PD-L1	NSCLC	Bintrafusp alfa + chemotherapy (docetaxel, platinum-based)	NCT02517398	Bintrafusp alfa induced promising efficacy and manageable tolerability in patients with NSCLC previously treated with platinum
	HPV-associated cancer		NCT02517398, NCT02517398, NCT04247282	Bintrafusp alfa showed clinical activity and manageable safety in HPV-associated cancers
	Esophageal cancer		NCT02517398, NCT02699515	Bintrafusp alfa showed clinical activity with manageable safety profile in patients with advanced esophageal adenocarcinoma
**Anakinra**				
IL-1	Multiple myeloma	Anakinra + immunomodulatory drug combination lenalidomide and dexamethasone	NCT00635154	Anakinra decreased the proliferative rates of tumor, leading to a chronic disease state with improved PFS in patients with multiple myeloma at high risk of progression to active myeloma
	CRC	Anakinra + 5-FU + bevacizumab		5-FU plus bevacizumab and anakinra had promising activity and a manageable safety profile in refractory metastatic CRC
**Bempegaldesleukin (NKTR-214)**				
IL-2	Melanoma	Bempegaldesleukin + nivolumab/pembrolizumab	NCT03635983, PIVOT-02	Bempegaldesleukin can be used in combination with nivolumab or pembrolizumab in patients with metastatic melanomas
	Urothelial carcinoma	Bempegaldesleukin + nivolumab	NCT02983045, PIVOT-02	Bempegaldesleukin combined with nivolumab is suggested as the first-line therapy for patients with metastatic urothelial carcinoma with manageable side effects
**Nemvaleukin alfa (LKS 4230)**				
**IL-2**	Ovarian cancer	Nemvaleukin alfa + pembrolizumab	NCT05092360	Under evaluation for the efficacy and safety as monotherapy and combination therapy with pembrolizumab in patients with platinum-resistant ovarian cancer
**CNTO 328**				
**IL-6**	Multiple myeloma	Siltuximab + bortezomib-melphalan-prednisone (VMP)	NCT00911859	The addition of siltuximab to the bortezomib-melphalan-prednisone (VMP) regimen did not improve the complete response rate or long-term outcomes of MM patients
	Prostate cancer	Siltuximab + mitoxantrone/prednisone	SWOG S0354	Siltuximab was well tolerated and improved clinical outcomes, leading to a PSA response rate of 3.8% and a stable disease rate of 23% in patients with castration-resistant prostate cancer
**Tocilizumab**				
**IL-6R**	Ovarian cancer	Tocilizumab + carboplatin/doxorubicin	NCT01637532	Tocilizumab at 8 mg/kg combined with carboplatin/doxorubicin chemotherapy is feasible and safe for the treatment of ovarian cancer
**Pegilodecakin (LY3500518)**				
IL-10	Solid tumors	Pegilodecakin + chemotherapies or anti-PD-1 blockade	NCT02009449	Pegilodecakin was used as monotherapy and in combination with chemotherapies or anti-PD-1 blockade to treat tumors such as melanoma, NSCLC, CRC, and pancreatic cancer
**Chemokine-directed therapies**				
**Carlumab**				
CCL2	Prostate cancer			Carlumab could be safely administered in patients with metastatic CRPC, but failed to demonstrate significant antitumor activities as a single agent
**PF-04136309**				
CCR2				
	Pancreatic cancer	PF-04136309 + chemotherapy (gemcitabine plus nab‐paclitaxel)	NCT02732938	PF-04136309 in combination with nab-paclitaxel plus gemcitabine may induce pulmonary toxicity, with no significant superior efficacy signal over nab-paclitaxel and gemcitabine

CML, chronic myeloid leukemia; AML, acute myeloid leukemia; HCC, hepatocellular carcinoma; NSCLC, non-small cell lung cancer; ICC, intrahepatic cholangiocarcinoma; SCCHN, squamous cell carcinoma of head and neck; ALL, acute lymphocytic leukemia; CNS, central nervous system; SCLC, small cell lung cancer; PDAC, pancreatic ductal adenocarcinoma; RT, radiation therapy; EGFR, epidermal growth factor receptor; TKIs, tyrosine kinase inhibitors; PSA, prostate-specific antigen

### Non-steroidal anti-inflammatory drugs (NSAIDs)

With the advent of aspirin in the 1990s, the application of NSAIDs has been extended to the treatment of pain, fever, and other inflammatory processes. Multiple studies have addressed the preventative effect of NSAIDs on cancer, leading to reduced incidence of colorectal [277], breast [278], and esophageal cancer [279]. In a randomized clinical trial, daily administration of aspirin effectively prevented adenoma growth in patients with familial adenomatous polyposis [280, 281]. Another clinical trial demonstrated that aspirin decreased the recurrence rates of colorectal adenomas and the incidence of CRC in patients with hereditary Lynch syndrome [277]. A multicenter, randomized controlled clinical trial (AspECT) aimed to investigate the long-term chemoprevention effect of esomeprazole proton-pump inhibitor (PPI) and aspirin, suggesting that the combination treatment of aspirin and esomeprazole significantly improved the clinical outcome of patients with Barrett's esophagus, thereby reducing the risk of esophageal cancer [282].

A major mechanism through which NSAIDs suppress carcinogenesis is the eicosanoid signaling. NSAIDs inhibit the cyclooxygenases (COX-1 and COX-2), but not the lipoxygenases. As the levels of PGE2 and COX-2 are often elevated in cancers such as CRC [283, 284], COX-2 inhibitors especially COXIBs (selective COX-2 inhibitors) were developed, with potent anti-inflammatory activities without affecting the physiological functions of COX-1 [285]. Thus, COXIBs are believed to cause fewer gastrointestinal side effects compared with non-selective NSAIDs and at the same time derive the same benefits [238]. In 1999, the Food and Drug Administration (FDA) approved the use of celecoxib, a COXIB, in patients with familial adenomatous polyposis [286].

Multiple clinical trials have evaluated the potential of celecoxib for the prevention and treatment of cancer patients. For instance, the concomitant use of celecoxib and chemotherapy (FOLFIRI regimen consisting of 5-flourouracil, leucovorin, irinotecan) may represent an effective and safe synergetic protocol for patients with metastatic CRC (NCT03645187) [287]. Celecoxib also demonstrates excellent efficacy in the prevention of colorectal adenomas (NCT00005094) [288]. The administration of celecoxib significantly reduced the occurrence of colorectal adenomas in patients receiving polypectomy (NCT00141193) [289]. Celecoxib has also been tested in synergy with PD-1 blockade toripalimab, which induced a high pathological complete response rate and an acceptable safety profile in patients with mismatch repair (MMR) deficient or microsatellite instability (MSI)-high CRC (NCT03926338) [290]. A meta-analysis further confirmed the potential of celecoxib-combined cancer therapy in improving clinical outcomes in several cancer types [291]. In patients with positive COX-2-positive gastric cancer, combination therapy of celecoxib and chemotherapy significantly improved disease-free survival (DFS), progression-free survival (PFS), and short-term clinical efficacy, without increasing the incidence of adverse events (AEs) [292]. In lung cancer, celecoxib at a maximal tolerated dose of 800 mg/d can be safely administered concurrently with thoracic radiotherapy and resulted in PFS rates of 66.0% at 1 year and 42.2% at 2 years [293]. In other phase II trials however, celecoxib treatment (NCT00300729) or adding celecoxib to concurrent chemoradiation (NCT01503385) did not improve survival of NSCLC patients [294, 295]. In a phase II trial, celecoxib induced favorable changes in serum biomarkers and cytology in women with increased risk for breast cancer [296]. Notably, the improvement of prognosis by celecoxib-based combination treatment is more prominent in patients with tumors expressing higher levels of COX-2 [297]. No statistical difference in AEs was identified between treatment group and control group, such as dysphagia, anxiety, dry mouth, and hair loss. Celecoxib treatment induced a significantly higher pathological complete response (pCR) rate in breast cancer patients with COX2-overexpressing tumors [298].

However, a recent clinical trial suggested that the addition of celecoxib to the standard adjuvant chemotherapy regime failed to bring more benefits to patients with stage III colon cancer (NCT01150045) [299]. Another study evaluated the efficacy of celecoxib as a combination partner for conventional therapy in ERBB2-negative breast cancer, which demonstrated no significant benefits from celecoxib in terms of DFS following 2-year treatments (NCT02429427) [300]. Moreover, some studies suggested that the addition of celecoxib to chemotherapy might adversely impact the prognosis of breast cancer patients, especially those with prostaglandin-endoperoxide synthase 2 (PTGS2) low tumors (NCT01041781) [301]. Such conflicting results likely reflect the impact of different treatment regimens or administration doses of celecoxib, and the expression profile of biomarkers in tumors. Thus, all the above factors should be taken into account to investigate the therapeutic potential of celecoxib. In addition, long-term use of NSAIDs including COXIBs at high doses may lead to severe cardiovascular side effects in patients, especially in those with a history of atherosclerotic heart disease [302]. One way to prevent or reduce these side effects would be the alternative targeting of the downstream PGE2 pathway. Some researchers have introduced natural compounds with known inhibitory activities on COX-2, such as natural phenols, flavonoids, stilbenes, terpenoids, quinones, and alkaloids [303].

### Antiviral therapies

#### Antihepatitis B virus (HBV) therapies

The majority of hepatocellular carcinoma (HCC) cases are associated with known risk factors, such as chronic hepatitis B virus infection. During chronic hepatitis B (CHB) infection, the immune response to persistent infection may cause chronic inflammation and hepatic fibrogenesis, leading to irreversible damage in the liver structure. The continuous replication of virus DNA and its integration into host genomes may cause genetic alterations, ultimately driving the carcinogenesis of hepatocytes [120]. On the other hand, viral proteins such as hepatitis B virus X protein may increase the sensitivity of the host to chemical carcinogens [304]. These preclinical studies have motivated the design of antiviral therapies in the treatment of HBV-related hepatocellular carcinoma.

The antiviral therapies aim to suppress HBV DNA replication, promote the serum conversion of hepatitis B e antigen (HBeAg), and attenuate the development of cirrhosis. Common antiviral drugs include the nucleoside and nucleotide analogs (NAs) and IFNs. Among them, the long-term administration of potent NAs with high barrier to resistance such as entecavir and tenofovir disoproxil, was recommended as first-line anti-HBV drugs in the clinical management consensus of CHB [305]. In a randomized controlled trial involving 299 centers in Asia, Europe, and North and South America with a 10 year of follow-up, patients treated with entecavir had a reduced risk of HBV-related events including HCC (NCT00388674) [306]. A nationwide population-based cohort study on CHB patients suggested that tenofovir treatment had lower incidence of HCC compared with entecavir treatment [307]. The superiority of tenofovir over entecavir in reducing HCC incidence in CHB patients was further confirmed in several other studies [303, 308]. However, some studies failed to identify clinically meaningful difference in the risk of liver-related events or deaths including HCC between entecavir- and tenofovir-treated cohorts, suggesting that the choice between tenofovir or entecavir should be based on patients’ tolerability (NCT019553458) [309, 310]. A recent study compared the long-term risk of tenofovir versus entecavir on HCC and intrahepatic cholangiocarcinoma (ICC) in CHB patients and suggested a comparable long-term risk between these two agents [311]. Recently, some antifibrotic Chinese herbs have been introduced to the antiviral therapy formulas for the treatment of CHB-related liver fibrosis. For instance the therapeutic potential of entecavir combined with Ruangan granule to reverse advanced liver fibrosis is currently being investigated in a number of clinical studies [312, 313].

#### Antihuman papillomavirus (HPV) therapies

Persistent HPV infection is a well-established risk factor for cervical cancer or precancerous cervical dysplasia [314, 315]. HPV proteins are implicated in the development of chronic inflammation [316]. The persistent HPV infection initiates a chain of reactions that regulate the secretion of inflammatory cytokines and immune cell infiltration [317]. For instance, the sustained elevation of systemic inflammatory cytokine levels was observed in older populations with chronic HPV infection [318], which potentially increased the risk for cervical cancer in this age group [319, 320].

The efficacy of HPV vaccines against cervical precancerous lesions has been confirmed by multiple large-scale reports. The population-based vaccination not only decreased the infection rates of HPV, but also the incidence of cervical intraepithelial neoplasia in women aged 20–24 years [321]. Recent results from a nationwide clinical study suggested that the cumulative incidence of cervical cancer was dramatically reduced by approximately 50% in women received the quadrivalent HPV vaccine at 10–30 years of age [322]. Given that antiviral drugs that specifically target HPV infections are still lacking, increasing HPV vaccination coverage in the population would potentially facilitate cervical cancer occurrence [323]. The first-in-human clinical trial of Vvax001, an alphavirus-based vaccine against HPV, was conducted in patients with HPV-induced cancers to assess its immunological activity, safety, and tolerability. The preliminary results supported the therapeutic application of Vvax001 in patients with HPV-related malignancies [324]. Similarly, the long-term follow-up results from a randomized, double-blind, controlled trial demonstrated that the bivalent HPV vaccine was highly effective in preventing HPV 16/18-associated precancer, further supporting the possibility to prevent invasive cervical cancer [325]. Another randomized trial investigated the combinational efficacy of anti-PD-1 antibody nivolumab with ISA 101, a synthetic HPV-16 vaccine, in patients with HPV-16-positive cancer. The combination therapy has increased both overall response rates and survival compared with PD-1 blockade monotherapy (NCT02426892) [326].

### Cytokine- and chemokine-directed therapies

The intratumoral infiltration of leukocytes and their release of soluble factors are important parts of the cancer-associated inflammation. These secretory factors include inflammatory cytokines such as IL-6, TNF-α, and IL-1b which facilitate the proliferation and metastasis of tumor cells, and suppress antitumor immune responses. We herein describe the anticancer therapies targeting cytokines or chemokines involved in cancer-related inflammation.

#### IFN-α-directed therapies

During the past decades, the adjuvant IFN-α therapy was intensively studied for the treatment of pancreatic cancer, with markedly improved prognosis observed from several clinical trials [327–330]. IFN-α was initially used as adjuvant therapies for patients with high-risk melanoma, which improved both relapse-free survival (RFS) and OS in patients receiving surgical treatments [331]. Adjuvant treatment with IFN-α-2a could improve the DFS and potentially OS of melanoma, with no improvement in clinical outcomes by PEG-IFN over IFN (NCT00204529) [332]. Nevertheless, inconsistent data were reported by some clinical trials that IFN-α derived no apparent benefits on the OS of patients [333]. High-dose interferon (IFN) for 1 year (HDI) has been approved by the FDA as adjuvant therapy for melanoma. In Japanese populations, PEG IFN-α-2b was well tolerated and approved in 2015 as adjuvant therapy in patients with stage III malignant melanoma [334]. Though approved by FDA for the treatment of melanoma and RCC, recombinant IFN-α is currently not a mainstream option due to the high incidence of AEs [335, 336]. Long-term follow-up results from the randomized phase III trial EORTC 18991 suggested that adjuvant PEG-IFN-α-2b therapy was able to induce sustained improvement of RFS in stage III melanoma patients [337]. On the other hand, PEG-IFN-α-2b may also negatively impact the health-related quality of life (HRQOL) of patients [338]. A phase III trial S0008 compared the efficacy of HDI regimen with short-term biochemotherapy consisting of dacarbazine, cisplatin, vinblastine, IL-2, IFN-α-2b, and GCSF and reported significant improvement in RFS but no significant difference in OS [339]. The grade 3 and 4 adverse events occurred in 57% and 7% of HDI patients, compared with 36% and 40% in biochemotherapy patients. IFN-α is also frequently used as a combination partner for immunotherapies or target therapies. The combination of the BRAF inhibitor vemurafenib and PEG-IFN-α-2b was well tolerated in melanoma patients whose treatment response was correlated with IFNAR1 expression levels (NCT01959633) [340]. Previous data supported the prophylactic administration of PEG-IFN-α for leukemia patients during the treatment of peri-hematopoietic cell transplantation (HCT) to prevent leukemia relapse (NCT02328755) [341]. IFN-α treatment is an effective strategy for minimal residual disease (MRD)-positive leukemia patients receiving allogeneic hematopoietic stem cell transplantation (allo-HSCT) (NCT02185261) [342].

IFN-α is a promising combination therapy for target therapies and immune checkpoint inhibitors such as anti-PD-1 therapies [343]. The prolonged IFN-α treatment results in long-lasting complete responses and long-term outcome with acceptable toxicity in patients with metastatic RCC. Sorafenib, a kinase inhibitor drug approved for the treatment of primary kidney cancer, concurrently used with IFN-α has been proved safe and effective for metastatic RCC patients (UMIN000002466) [344]. Similarly, bevacizumab plus IFN led to superior benefits in terms of PFS and ORR in patients with metastatic RCC as compared with IFN monotherapy (CALGB 90206) [345]. Recent research has focused on the potential of IFN-α in combination with ICBs which may overcome the treatment resistance to ICBs [346]. In NSCLC patients treated with nivolumab, a significantly elevated level of peripheral IFN-α was observed in those with longer PFS, indicating the synergistic effect of regional IFN-α with anti-PD-1 therapy [347]. The combination of ipilimumab with high dose IFNα2b (HDI) demonstrated an acceptable toxicity profile and a promising tumor response in ICB naïve patients (no treatment history of ICB) [348, 349]. Another factor that limits the use of IFNs is the short half-life of IFNs which makes it difficult to deliver IFNs to tumor sites at sufficient concentrations. To solve this, IFNs conjugated to tumor-specific mAbs were developed. An early example is the anti-CD20-IFN-α2 conjugate which increased antibody-dependent cytotoxicity and overcame the resistance to anti-CD20 treatment alone in mouse models [350, 351]. In addition, the anti-VEGFR mAb-conjugated IFN-α could inhibit the angiogenesis and promote immune responses in CRC tumor models [352]. IL-4 fused to pseudomonas exotoxin represents another novel combination partner for IFNs, which was found to improve the OS of mice with ovarian cancer xenograft, potentially by activating the key mediators of apoptosis [353].

Given the potential antitumor activities of IFN-α described in previous literature, IFN-α is also used as an adjuvant in tumor vaccines such as DC vaccines, augmenting their efficacy in tumors [354, 355]. For instance, IFN-α-conditioned DCs significantly increased the number of tumor-specific CD8 + T cells with cytotoxic phenotypes than cytokine cocktail-mDCs in RCC patients [356]. In a phase I clinical study, IFN-DCs were well tolerated and included marked immunological responses in advanced melanoma patients [357]. More recently, IFN-DCs were used as a novel DC-based immunotherapy for non-Hodgkin lymphomas (NHL) [358].

#### TGF-β-directed therapies

Therapeutic approaches targeting TGF-β mainly include: (1) the small-molecule inhibitors of TGF-β receptor I (TGF-βRI) such as galunisertib; (2) anti-TGF-β mAbs such as fresolimumab; (3) antagonistic mAbs targeting TGF-βR and TGF-β ligand traps [359]. Fresolimumab (GC1008) is a TGF-β-blocking antibody that neutralizes all mammalian active isoforms of TGF-β and was reported to induce stable disease in 6 out of 29 melanoma patients [360]. In patients with advanced melanoma and RCC, fresolimumab displayed preliminary antitumor efficacy and acceptable safety profile at multiple doses [360]. For patients with advanced malignant melanoma and RCC, Fresolimumab was safe and displayed preliminary antitumor efficacy (NCT00356460) [360]. A recent study examined the efficacy and immune effects of fresolimumab in metastatic breast cancer patients during radiotherapy treatment, where a favorable systemic immune response was observed. Notably, fresolimumab improved the OS of patients in a dose-dependent manner, with longer median OS observed in those treated at higher dose [361].

Galunisertib is a TGF-β1 receptor type I inhibitor and was intensively studied for the treatment of HCC and pancreatic cancer. The combination of galunisertib and sorafenib demonstrated improved prognosis of HCC, with neutropenia, fatigue, anemia, increased bilirubin, hypoalbuminemia, and embolism being the most common treatment-related AEs. (NCT01246986) [362, 363]. The galunisertib–gemcitabine combination improved OS in patients with unresectable pancreatic cancer with minimal added toxicity [364]. Galunisertib co-administered with durvalumab was tolerable, but with limited clinical activity which required the selection of predictive biomarkers for TGF-β inhibition in pancreatic cancer patients (NCT02734160) [365]. In a phase Ib/II study, galunisertib combined with checkpoint inhibitor nivolumab was well tolerated in NSCLC (NCT02423343) [366]. In this phase of the trial, the most frequent AEs were pruritus, fatigue, and decreased appetite. In addition, the addition of galunisertib to neoadjuvant chemoradiotherapy was well tolerated and improved the complete response rate in patients with rectal cancer (NCT02688712) [367].

PF-03446962 is a monoclonal antibody (mAb) targeting activin receptor like kinase-1 (ALK1), a TGF-βR subtype, which showed limited activity in urothelial carcinoma and is thus not recommended as monotherapy [368]. A phase I study reported manageable safety and pharmacokinetic profiles with promising clinical activity, supporting further evaluation of PF-03446962 in patients with HCC and other solid malignancies (NCT00557856) [369]. However, several other clinical trials failed to identify improvement of objective responses in patients with HCC, RCC, NSCLC, and malignant pleural mesothelioma [369–371]. More recently, the combination of regorafenib and PF-03446962 was found to cause unacceptable toxicity with limited clinical activity in patients with refractory metastatic CRC [372]. Thus, PF-03446962 has not been developed further.

Based on the observation that TGF-β signaling was associated with treatment resistance to anti-PD-L1 therapies, a novel dual-targeting agent bintrafusp alfa was developed. Bintrafusp alfa is a bifunctional fusion protein consisting of the extracellular domain of the TGF-βRII receptor and a PD-L1-blocking immunoglobulin G1 (IgG1) mAb [373].

An expansion cohort of a phase trial suggested that bintrafusp alfa induced encouraging efficacy and manageable tolerability in patients with NSCLC previously treated with platinum (NCT02517398) [374]. Bintrafusp alfa has demonstrated potent clinical activity with manageable safety in patients with HPV-associated cancer (NCT02517398, NCT02517398, NCT04247282) and esophageal adenocarcinoma (NCT02517398, NCT02699515) [375–379]. Moreover, the simultaneous inhibition of TGF-β and PD-L1 by bintrafusp alfa could synergize with radiotherapy in radioresistant tumor models [380]. These results collectively support the clinical translation of this dual-targeting agent in treating therapy-resistant tumors, with minimal damage to normal tissues.

#### IL-1-directed therapies

In the clinical setting, many NSCLC tumors displayed low PD-L1 expression, which requires other treatment options to improve the efficacy of ICBs. As aforementioned, the elimination of MDSCs in the TME by inhibiting the IL-1 pathway is a potential strategy to overcame tumor resistance to immunotherapies such as immune checkpoint blockades [381],which has been evaluated in different models. Anti-IL-1β mAbs could enhance the efficacy of PD-1 blockades against breast cancer [163]. In a RCC mouse model, the combination of IL-1β blockade with either anti-PD-1 or tyrosine kinase inhibitors achieved greater antitumor efficacy than either monotherapy [382].

Canakinumab is an anti-IL1β mAb that has been approved for use in a variety of immune-related disorders. Clinical inhibition of IL-1β by canakinumab in lung cancer was first reported in a phase III study, the Canakinumab Anti-inflammatory Thrombosis Outcomes Study (CANTOS) [383]. In this trial, canakinumab reduced both the occurrence and mortality of lung cancer, providing the first rationale for the assessment of canakinumab use in lung cancer patients [384]. Though with less lung cancer mortality, canakinumab 300 mg group had higher incidence of fatal infections or sepsis than the placebo group. CANOPY-N is a randomized phase II trial investigating the efficacy of combination therapy with canakinumab and pembrolizumab as neoadjuvant treatment in patients with non-small cell lung cancer (NSCLC) [385]. Later evidence suggested that blocking IL-1β with canakinumab may be a preventive approach for individuals with high risks for KM-LUAD [386].

Anakinra is a human anti-IL-1R1 antibody and has been approved by the FDA for the treatment of rheumatoid arthritis. Anakinra has also been used for the treatment of several cancers [387–390]. Preclinical studies reported that gemcitabine and 5-fluorouracil (5-FU) could promote IL-1β production in a T-cell lymphoma-bearing mouse model, which restrained the efficacy of chemotherapeutic agents [391]. Thus, anakinra can be used as an adjunctive therapy to enhance the efficacy of chemotherapy of 5-FU. In the clinical context, the combination of chemotherapy with 5-FU, anakinra, and bevacizumab led to an increased median PFS and OS of patients with metastatic CRC with minimum AEs [392]. In patients with multiple myeloma at high risk of progression to active myeloma, treatment with anakinra decreased the proliferative rates of tumor, leading to a chronic disease state with improved PFS (NCT00635154) [393].

#### IL-2-directed therapies

IL-2 is a key growth factor for CD4 + T cells and NK cells and is involved in the regulation of T cell proliferation, survival, and differentiation [394–396]. IL-2 has been described as a immunostimulant, and its anticancer activities have been studied for more than 30 years [397]. The intravenous administration of recombinant IL-2 was approved by the FDA for the treatment of metastatic RCC in 1992 and melanoma in 1998. Though IL-2 treatment could induce durable response in melanoma and RCC patients [398], the short half-life of IL-2 requires a therapeutic schedule with an 8-h interval. Moreover, a high incidence of severe AEs including vascular leak syndrome and cardiac toxicities was frequently reported due to the high dose of IL-2 to reach its efficacy [399]. IL-2 was also shown to promote the activities of immunosuppressive Tregs, which casted doubt on the antitumor role of IL-2 [399]. The impact of IL-2 on Tregs might be attributed to the constitutive expression of IL-2 receptor on Tregs. This receptor consists 3 subunits (IL-2Rαβγ) and has higher affinity to IL-2 compared with those expressed on CD8 + T cells, memory T cells, and NK cells which lack the α subunit [400].

The differential expression of IL-2 receptors has motivated the design of IL-2R agonists that selectively activate the IL-2Rβγ complex on immunostimulatory immune cells. A PEGylated form of IL-2, bempegaldesleukin (NKTR-214/BEMPEG) preferentially interacts with the β subunit of IL-2R, specifically stimulating the antitumor activities of CD8 + T cells and NK cells [401]. Multiple clinical studies have identified bempegaldesleukin as a promising agent in reducing tumor volumes in pre-treated melanoma and RCC [402]. Bempegaldesleukin has also been investigated as a combination partner for nivolumab, which yielded objective response rates (ORRs) of approximately 33–75% in patients with melanoma, RCC, NSCLC, or triple-negative breast cancer (TNBC) [403]. A number of clinical trials are ongoing to assess the safety and clinical benefits of bempegaldesleukin when combined with pembrolizumab in patients with metastatic melanoma (NCT03635983) [404]. Bempegaldesleukin is also suggested to be used in combination with nivolumab as the first-line therapy for patients with metastatic urothelial carcinoma (NCT02983045) or metastatic melanoma (PIVOT-02), with manageable side effects [405, 406]. Nemvaleukin alfa (nemvaleukin, ALKS 4230) is a novel engineered forms of IL-2 that selectively binds to the IL-2R on antitumor CD8 + T cells and NK cells with minimal effect on immunosuppressive Tregs [325]. In a novel SCLC murine model, the mouse version of nemvaleukin (mNemvaleukin) significantly inhibited murine SCLC tumor growth and improved mouse survival, supporting the evaluation of nemvaleukin alone or in combination with chemotherapy in clinical trials [407]. Ongoing clinical trials such as ARTISTRY-7 trial compared efficacy and safety of nemvaleukin as monotherapy and combination therapy with pembrolizumab in patients with platinum-resistant ovarian cancer (NCT05092360) [408–410].

In addition to engineered IL-2 that activates the IL-2Rβγ complex, another therapeutic strategy is to target IL-2α (CD25) and thus deplete the immunosuppressive Tregs. Earlier studies reported that the intravenous infusion of daclizumab monotherapy induced a significant and persistent decrease in CD25 + FOXP3 + Tregs in peripheral blood of breast cancer patients [411]. This result was further confirmed in patients with glioblastoma [412] and metastatic melanoma [413]. More recently, preclinical evidence suggested that the antihuman CD25 mAb (RG6292) efficiently induced Treg depletion and held great potential for the anticancer treatments in combination with ICBs [414]. It was later identified that the combination of anti-CD25 antibodies and anti-PD1 antibodies markedly promoted the tumor rejection induced by CD25 antibodies [415]. Moreover, the inhibitory effect of anti-CD25 antibodies in combination with radiotherapy was assessed on the local tumor growth and hepatic metastasis rectal cancer, which suggested that the depletion of Tregs could improve the antitumor effect of radiotherapy plus and produce an abscopal effect [416]. These data collectively support the clinical evaluation of RG6292 incorporating non-IL-2 blocking anti-CD25 antibodies [414].

#### IL-6-directed therapies

The therapeutic targeting of IL-6 cytokine family members includes the direct blocking of cytokines or their receptors by monoclonal antibodies and small molecules that inhibit the receptor signaling of gp130 and JAK–STAT pathway. These therapeutic strategies are best represented by the monoclonal antibodies targeting IL-6.

IL-6 has long been identified as a key growth factor for myelomas. Between in 1988 and 1989, three laboratories independently reported the promoting effect of IL-6 on the proliferation of in human multiple myeloma (MM) [417]. In 1991, researchers found that the sequential injections of mouse anti-IL-6 antibodies led to reduced MM cell proliferation [418]. Since then, IL-6 has been intensively investigated as a therapeutic target for MM in a number of clinical trials [419]. However, results form later clinical trials were unsatisfactory, and anti-IL6 mAb has thus not been approved for MM to date [420, 421]. Siltuximab (CNTO 328) is an anti-interleukin-6 chimeric mAb, the addition of which to the bortezomib-melphalan-prednisone (VMP) regimen did not improve the complete response rate or long-term outcomes of MM patients (NCT00911859) [421]. A phase I/II study reported that siltuximab stabilized disease in > 50% of progressive metastatic RCC patients [422]. Results from SWOG S0354 trial suggested that siltuximab resulted in a prostate-specific antigen (PSA) response rate (defined as 50% reduction) of 3.8% and a stable disease rate of 23% in patients with castration-resistant prostate cancer (CRPC) [423]. For CRPC patients with prior chemotherapy treatment, siltuximab plus mitoxantrone/prednisone (M/P) was well tolerated and improved clinical outcomes [424].

Due to the elevation in systemic IL-6 levels caused by anti-IL-6 mAbs [425], some alternative IL-6-directed therapies have been developed such as functional blocking of IL-6 receptors (IL-6R). Administration of IL-6R inhibitor tocilizumab at 8 mg/kg combined with carboplatin/doxorubicin chemotherapy is feasible and safe for the treatment of ovarian cancer (NCT01637532) [426]. Unfortunately these modalities are not further investigated in the treatment of cancer patients. One possible explanation is that cytokine receptors such as IL-6Rα may interact with more than one cytokine. The therapeutic targeting of IL-6R may thus result in unexpected AEs compared with the inhibition of an individual cytokine.

#### IL-10-directed therapies

IL-10 was initially identified as an immunosuppressive cytokine [427], but recent researches have also identified the antitumor effect of IL-10 by stimulating CD8 + T cell in tumor models [428, 429]. As aforementioned, the dual role of IL-10 in tumor progression may vary according to tumor types, or the stage of T cells that respond to IL-10. Though tumor vaccines are known to upregulate tumor-specific CD8 + T cells, they often fail to increase the number of tumor reactive T cells in the TME. An earlier study suggested that the sustained treatment with IL-10 could induce the activation and expansion of tumor-resident CD8 + T cells in mouse tumor models [428]. IL-10-induced tumor rejection could not be impaired by the inhibition of T-cell trafficking from lymphoid organs, indicating its activation on tumor-resident CD8 + T cells. Moreover, the antitumor immune response is mediated directly through expansion of intratumoral CD8 + T cells, whereas the expression of IL-10 receptors on other cells was not necessary for such tumor rejection.

A series of trials have been conducted using the PEGylated recombinant human IL-10 (AM0010, pegilodecakin) in patients with advanced-stage solid tumors [430]. Pegilodecakin is a long-acting, PEGylated version of IL-10 which was found to induce the expression of IFN-γ and granzymes in tumor-infiltrating CD8^+^ T cells, thereby increasing the number and enhancing the activities of CD8 + T cells. In a multi-institution trial (NCT02009449), pegilodecakin was used as monotherapy and in combination with chemotherapies or anti-PD-1 blockade to treat tumors such as melanoma, NSCLC, CRC, and pancreatic cancer [431]. The safety profile of pegilodecakin significantly differs from other interleukin therapies with frequent occurrence of the cytokine release syndrome [432]. The most frequent treatment-related AEs of pegilodecakin are thrombocytopenia and anemia. The occurrence of anemia might be attributed to the increased phagocytosis of aging red blood cells by activated macrophages [433]. Given that pegilodecakin monotherapy could increase the number of activated infiltrating CD8 + T cells, pegilodecakin is particularly applicable for patients with low T cell-infiltrated tumors prior to therapy [434] and those with tumors refractory to standard therapies [431].

Pegilodecakin was further evaluated in combination with anti-PD-1 inhibitors nivolumab or pembrolizumab for patients with melanoma, NSCLC, or RCC [435]. In the phase II CYPRESS 1 and CYPRESS 2 trials, the concomitant use of pegilodecakin and PD-1 blockades was tested in patients with NSCLC. Unfortunately no significant synergistic effects were observed with the drug combinations relative to the respective PD-1 blockade alone [435–437]. More recently, results from a phase I/Ib multi-cohort IVY study reported that pegilodecakin and PD-1 blockades showed promising clinical activity and consistent safety profile as previously reported [438]. Pegilodecakin also enhanced the treatment response of patients with heavily pretreated RCC to anti-PD-1 therapies [438]. Though promising antitumor efficacy was reported in patients with metastatic PDAC [439], the addition of pegilodecakin to the second-line FOLFOX chemotherapy failed to improve either PFS or OS in a phase III trial [440].

#### CCL2/CCR2 axis-directed therapies

As a potent proinflammatory chemokine signaling, the CCL2/CCR2 axis is important for the recruitment and survival of myeloid cells including inflammatory monocytes, TAMs, and MDSCs [441]. The inhibition of the CCL2/CCR2 axis was thus investigated as a therapeutic strategy to modify the immunosuppressive TME and activate antitumor immunity. The first-in-human clinical trial of carlumab (CNTO 888), a human anti-CCL2 mAb, identified transient free CCL2 suppression and antitumor efficacy in patients with solid tumors [442]. In a phase II study, carlumab could be safely administered in patients with metastatic CRPC, but failed to demonstrate significant antitumor activities as a single agent [443]. Later in another phase I trial (NCT01204996), carlumab was tested in combination with four chemotherapy regimens in patients with solid tumors. Though carlumab was well tolerated in combination with standard chemotherapies, with the most common drug-related grade 3/4 AEs being neutropenia for docetaxel and gemcitabine, long-term tumor responses were not identified in tested patients [444].

Given the suboptimal clinical efficacy of CCR2 inhibitors as monotherapy, the therapeutic potential of CCR2 inhibitors to work in synergy with chemotherapies and immune checkpoint inhibitors was then evaluated. PF-04136309 is a small-molecule CCR2 inhibitor which was mainly studied in the context of pancreatic cancer. In a phase I trial, the targeting of TAMs with PF-04136309-FOLFIRINOX combination was safe and tolerable in patients with borderline resectable and locally advanced pancreatic cancer [445]. Unfortunately, PF-04136309 combined with nab-paclitaxel plus gemcitabine resulted in synergistic pulmonary toxicity, with no superiority over in terms efficacy in PDAC patients (NCT02732938) [446]. CCR2i is a competitive binding inhibitor with a selective and high affinity for the binding pocket of CCR2 and, when combined with an immune checkpoint inhibitor, could suppress tumor growth of cutaneous T-cell lymphomas [447]. BMS-687681, a dual inhibitor targeting CCR2 and CCR5, was used as a prolonged treatment following αPD-1 and radiotherapy in PDAC mouse models, which conferred better antitumor efficacy than other tested combination regimes [448, 449]. Notably, this combination treatment altered the TME by increasing intratumoral effector and memory T cell infiltration and reducing the infiltration of Tregs, M2 TAMs, and MDSCs. The simultaneous administration of CTLA-4 blockades and CCR2 inhibitors led to potent antitumor immunity, further supporting the clinical translation of CCR2/5i in combination with ICIs [450].

### Natural anti-inflammatory therapies

Many natural compounds that derive form natural resources such as plants are currently used as therapeutic drugs in cancer. A well-known example is curcumin, also known as diferuloylmethane. Curcumin is the key component of turmeric and has long been used for multiple medical purposes since ancient times [451]. Curcumin is involved in a series of inflammatory pathways implicated in tumorigenesis and has been characterized as a potent antitumor agent. In a systematic review based on multiple databases, analyses on clinical trails between 1980 and 2019 showed that dietary curcumin could reduce the level of C-reactive protein, IL-6, TNF-α, and MCP-1, and increase the level of IL-10, providing evidence for the anti-inflammatory effect of curcumin in chronic inflammation [452]. Notably, the intended use of curcumin was approved by the FDA as “Generally Recognized As Safe” (GRAS) [453].

Curcumin not only reduces cancer risks, but also increases the sensitivity of tumors to chemotherapy and radiotherapy [454]. In light of the frequent AEs associated with 5FU-based or oxaliplatin-based chemotherapy in advanced CRC patients, natural compounds such as curcumin are used as adjuncts to currently available treatment options. In a phase I trial, curcumin administration for up to 4 months was well tolerated in CRC patients [455]. In a phase II randomized controlled trial, curcumin was a safe and tolerable adjunct to folinic acid/5-fluorouracil/oxaliplatin chemotherapy (FOLFOX) chemotherapy in patients with metastatic CRC [456]. In breast cancer, curcumin reduced the paclitaxel (PTX)-induced EGFR, ERK1/2, and AKT expression and could thus synergize with PTX in suppressed tumor growth [457]. Moreover, the increased apoptosis of breast cancer cells induced by PTX-curcumin combination may be mediated via the upregulation of activated caspase 3 and PARP cleavage [458]. Other natural compounds such as quercetin and resveratrol have demonstrated preclinical antitumor efficacy, but no clearly established results were reported from human trials (NCT01538316, NCT01879878, NCT00003365).

Resveratrol is another anti-inflammation agent that inhibits the release of proinflammatory cytokines of T cells [459]. Th17 is a predominant T cell subset targeted by resveratrol. By activating sirtuin-1, resveratrol reduces the acetylation of p65/relA, ultimately suppressing the activation of NF-kB pathway. Moreover, activated sirtuin-1 may also cause STAT3 deacetylation, impeding the activation of retinoid orphan receptor gamma t (RORγt) and the production of IL-17 [460]. RORγt suppresses Th1 differentiation and thus switches the Th1/Th2 balance toward anti-inflammatory (Th2) and immunoregulatory (Treg) responses. In addition, resveratrol also leads to an increased level of anti-inflammatory macrophages (M2). Resveratrol impedes LPS-induced macrophage activation by inhibiting NF-kB and COX-2 signaling and inflammasome activation [459]. In a clinical study, daily consumption of resveratrol induced substantial antitumor effect in 20 patients with colorectal cancer, suggesting the potential of resveratrol as a chemopreventive drug in cancer.

## Conclusions and future perspectives

In this review, we described the key inflammatory mediators in cancer. Inflammation, particularly the chronic inflammation, may serve as tumor initiators and promote tumor survival, invasion, and metastasis. It is thus conceivable that targeting inflammation mediators may facilitate the treatment of cancer patients. On one hand, inflammation-directed therapies aim to increase the tumor-killing capability by activating the anticancer immune cells. On the other hand, they may also reshape the TME by altering the immunosuppressive phenotypes of immune cells.

To date, a wide array of inflammation-directed therapies has been developed and is under evaluation both preclinically and clinically in cancer models. With the advances outlined herein, some anti-inflammatory approaches have proven rather effective in cancer prevention and treatment, providing solid scientific rationale for further development of such strategies. Moreover, some inflammatory responses following cancer therapies would confer residual cancer cells with resistance to subsequent treatments. Immunotherapies induce durable responses in only a small subset of patients, with the majority of patients eventually experiencing primary or acquired therapy resistance. Treatment resistance to immunotherapies is often attributed to the presence of proinflammatory and immunosuppressive TME [461]. One such example is the use of anti-CTLA-4 therapies that are related to incidence of colitis and hypophysitis [462], and anti-PD-1 therapies are associated with thyroiditis [463]. Thus, the addition of anti-inflammatory therapies into cancer treatment regimes would yield better clinical responses in some clinical cases.

The initial aim of anti-inflammatory therapies is to suppress the protumoral inflammation and at the same time activate antitumor immune response. Unlike therapies that target specific tumor markers, biomarkers for the selection of anti-inflammatory therapies are lacking. Intrinsic differences of patients such as age, and tumor molecular profile would affect the therapeutic response to inflammation-directed treatments. Thus, high-resolution methods such as multiomics, single-cell, and spatial analyses are recommended to facilitate medical decision and to predict the therapeutic response to inflammation-directed therapies. In addition, it still remains challenging to maintain the balance of inflammation in immune system. The heterogeneity and plasticity of the TME also pose challenges to inflammation-directed therapies by targeting a single molecule or immune cell type. For example, the disrupted feedback loops by targeting one inflammatory cytokine may lead to the compensatory activation of its involved pathways. Future studies are warranted to investigate the combination of inflammation-directed therapies and other treatment options for cancer, facilitating the design of safe and personalized treatment.

## Data Availability

The materials supporting our conclusion of this review are included within the article.

## Abbreviations

TME: Tumor microenvironment
ROS: Reactive oxygen species
TNF-α: Tumor necrosis factor-α
MIF: Migration inhibitory factor
TAMs: Tumor-associated macrophages
TANs: Tumor-associated neutrophils
DCs: Dendritic cells
MDSCs: Myeloid-derived suppressor cells
MMP: Matrix metallopeptidase
IFN: Interferon
TGF-β: Transforming growth factor-beta
CXCL: C-X-C motif chemokine ligand
ANG1: Angiopoietin-1
NETs: Neutrophil extracellular traps
EMT: Endothelial-to-mesenchymal transition
GM-CSF: Granulocyte–macrophage colony-stimulating factor
NK: Natural killer
ECM: Extracellular matrix
Tregs: Regulatory T cells
Th17: T helper 17
FLT3: Fms-related tyrosine kinase receptor 3
TAAs: Tumor-associated antigens
ICD: Immunogenic cell death
DAMPs: Damage-associated molecular patterns
ER: Endoplasmic reticulum
PRRs: Pattern recognition receptors
M-MDSCs: Monocytic-myeloid-derived suppressor cells
PBMC: Peripheral blood mononuclear cells
NO: Nitric oxide
TCR: T cell receptor
ICB: Immune checkpoint blockade
CSF-1R: Colony-stimulating factor-1 receptor
PrP: Prion protein
PAF: Platelet-activating factor
TIMPs: Tissue inhibitors of MMPs
HNSCC: Head and neck squamous cell carcinoma
IL: Interleukin
LDL-C: Low-density lipoprotein cholesterol
KM-LUAD: K-ras-mutant lung adenocarcinoma
Breg: Regulatory B
PDAC: Pancreatic ductal adenocarcinoma
NF-κB: Nuclear factor kappa B
LIF: Leukemia inhibitory factor
OSM: Oncostatin M
CNTF: Ciliary neurotrophic factor
CT-1: Cardiotrophin-1
CLC: Cardiotrophin-like cytokine
CDK: Cyclin-dependent kinase
XIAP: X-linked inhibitor of apoptosis protein
JAK: Janus kinase
STAT: Signal transducer and activator of transcription
PUFAs: Polyunsaturated fatty acids
COX: Cyclooxygenase
LOX: Lipoxygenase
PGs: Prostaglandins
LXs: Lipoxins
mPGES-1: Microsomal PGE2 synthase 1
LT: Leukotriene
CRC: Colorectal cancer
IBD: Inflammatory bowel disease
CAC: Colitis-associated CRC
NSAIDs: Non-steroidal anti-inflammatory drugs
DFS: Disease-free survival
PFS: Progression-free survival
AEs: Adverse events
MMR: Mismatch repair
MSI: Microsatellite instability
HCC: Hepatocellular carcinoma
HBV: Hepatitis B virus
NAs: Nucleotide analogs
RFS: Relapse-free survival
NHL: Non-Hodgkin lymphomas
5-FU: 5-Fluorouracil
MM: Multiple myeloma
